# Greenhouse Gas Emissions and Lateral Carbon Dynamics at an Eroding Yedoma Permafrost Site in Siberia (Duvanny Yar)

**DOI:** 10.1111/gcb.70071

**Published:** 2025-02-14

**Authors:** Kirsi H. Keskitalo, Lisa Bröder, Dirk J. Jong, Paul J. Mann, Tommaso Tesi, Anna Davydova, Nikita Zimov, Negar Haghipour, Timothy I. Eglinton, Jorien E. Vonk

**Affiliations:** ^1^ Department of Geography and Environmental Sciences Northumbria University Newcastle Upon Tyne UK; ^2^ Department of Earth Sciences Vrije Universiteit Amsterdam Amsterdam the Netherlands; ^3^ Department of Earth Sciences Swiss Federal Institute of Technology Zürich Switzerland; ^4^ National Research Council, Institute of Polar Sciences in Bologna Bologna Italy; ^5^ Pacific Institute for Geography, Far East Branch Russian Academy of Sciences, Northeast Science Station Cherskiy, Republic of Sakha, Yakutia Russia; ^6^ Laboratory of Ion Beam Physics Swiss Federal Institute of Technology Zürich Switzerland

**Keywords:** CH_4_, CO_2_, incubation, riverbank erosion

## Abstract

Rapid Arctic warming is accelerating permafrost thaw and mobilizing previously frozen organic carbon (OC) into waterways. Upon thaw, permafrost‐derived OC can become susceptible to microbial degradation that may lead to greenhouse gas emissions (GHG), thus accelerating climate change. Abrupt permafrost thaw (e.g., riverbank erosion, retrogressive thaw slumps) occurs in areas rich in OC. Given the high OC content and the increase in frequency of abrupt thaw events, these environments may increasingly contribute to permafrost GHG emissions in the future. To better assess these emissions from abrupt permafrost thaw, we incubated thaw stream waters from an abrupt permafrost thaw site (Duvanny Yar, Siberia) and additionally, waters from their outflow to the Kolyma River. Our results show that CO_2_ release by volume from thaw streams was substantially higher than CO_2_ emissions from the river outflow waters, while the opposite was true for CO_2_ release normalized to the suspended sediment weight (gram dry weight). The CH_4_ emissions from both thaw streams and outflow waters were at a similar range, but an order of magnitude lower than those of CO_2_. Additionally, we show that nearshore riverbank waters differ in their biogeochemistry from thaw streams and Kolyma River mainstem: particles resemble thaw streams while dissolved fraction is more alike to the Kolyma River thalweg. In these waters dissolved OC losses are faster than in the river thalweg. Our incubations offer a first insight into the GHG release from permafrost thaw streams that connect exposed and degrading permafrost outcrops to larger river systems.

## Graphical abstract

Rapid warming of the Arctic is accelerating permafrost (frozen ground) thaw and releasing previously frozen organic matter into streams and rivers. At an abruptly thawing permafrost site in Siberia (Duvanny Yar), we show that permafrost thaw streams and their outflow waters to the Kolyma River release greenhouse gases to the atmosphere. Additionally, our data show that biogeochemistry and degradation of organic matter in outflow waters differs from both thaw streams and the mainstem of the Kolyma River.

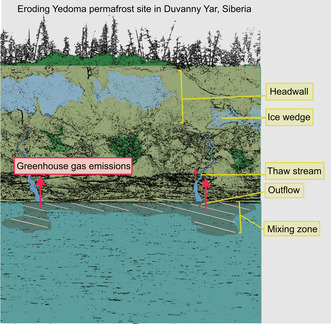

## TIIVISTELMÄ

Arktisten alueiden nopea lämpeneminen kiihdyttää ikiroudan sulamista ja voi vapauttaa aiemmin jäädyksissä ollutta orgaanista hiiltä virtavesiin. Sulamisen seurauksena ikiroudasta peräisin oleva orgaaninen hiili voi altistua mikrobien hajotusprosesseille ja johtaa kasvihuonekaasupäästöihin. Äkillisesti sulavat ikirouta‐alueet (esim. joenvarsien eroosio) varastoivat paljon orgaanista hiiltä. Orgaanisen hiilen määrän ja ikiroudan äkillisten sulamistapahtumien määrän kasvaessa, nämä alueet voivat vaikuttaa yhä enenevässä määrin kasvihuonekaasujen kokonaispäästöihin ikirouta‐alueilta. Jotta voisimme paremmin ymmärtää äkillisesti sulavien ikirouta‐alueiden kasvihuonekaasupäästöjä, inkuboimme äkillisestä ikiroudan sulamisesta syntyneiden sulavesipurojen vesiä (Duvanny Yar ‐alue, Siperia) sekä sulavesipurojen Kolyma‐jokeen valuvia ulosvirtausvesiä. Tuloksemme osoittavat, että sulavesipurojen hiilidioksidipäästöt tilavuuden mukaan laskettuna olivat huomattavasti korkeammat kuin ulosvirtausvesien päästöt, kun taas sedimenttien painoon (grammaa kuivapainona) suhteutettuna ulosvirtausvesien päästöt olivat korkeammat. Metaanipäästöt olivat samaa suurusluokkaa niin sulavesipuroissa kuin ulosvirtausvesissä, tosin huomattavasti matalammat kuin hiilidioksidipäästöt. Lisäksi tuloksemme osoittavat että ulosvirtausvedet eroavat biogeokemialtaan sekä sulavesipuroista että Kolyma‐joen syvänneurasta (ts. virtaveden syvin kohta): partikkelit muistuttavat sulavesipuroja kun taas liuenneet aineet Kolyma‐joen syvänneuraa. Inkubaatiokokeidemme aikana näissä ulosvirtausvesissä liuenneen orgaanisen hiilen määrä väheni nopeammin kuin Kolyma‐joen syvänneurassa. Inkubaatiokokeemme tulokset tarjoavat ensimmäisen katsauksen kasvihuonekaasupäästöihin sulavesipuroista, jotka yhdistävät äkillisesti sulavat ikirouta‐alueet suurempiin virtavesiin.

## 1 Introduction

The Arctic is warming nearly four times faster than the global average rate (Rantanen et al. 2022), causing widespread permafrost thaw across northern regions. The decomposition of recently thawed permafrost organic carbon (OC) releases greenhouse gases (GHG) into the atmosphere contributing to enhanced climate warming (e.g., Schuur et al. 2015). Current projections of future permafrost GHG emissions are primarily based on models simulating gradual permafrost thaw features (i.e., active layer deepening), while abrupt permafrost thaw processes (e.g., retrogressive thaw slumps, thermokarst, and river bank erosion) are poorly represented in Earth System Models (Natali et al. 2021; Schädel et al. 2024). Despite this, bottom‐up estimates using a synthesis of observations and first‐order (numerical inventory) models suggest that abrupt permafrost thaw processes may release up to 40% of the GHG emissions from gradual permafrost thaw by 2300 and double the overall global warming potential (Turetsky et al. 2019, 2020).

Decomposition of permafrost‐derived OC at abrupt permafrost thaw sites has been addressed using different incubation methods: traditionally in filtered waters measuring the loss of dissolved OC (DOC; e.g., Littlefair and Tank 2018; Mann et al. 2014, 2015; Vonk et al. 2015; this study) and more recently in whole‐water samples (i.e., including particles) using dissolved O_2_ as a proxy for microbial degradation (Shakil et al. 2022; this study). Furthermore, several studies (mostly using soil samples) have analyzed CO_2_ and CH_4_ from headspace (e.g., Dutta et al. 2006; Knoblauch et al. 2013, 2021; Lee et al. 2012; Tanski et al. 2019, 2021; this study), or directly measured CO_2_ and CH_4_ in the field (i.e., gas chamber method) to constrain GHG emissions from abrupt permafrost sites (e.g., Abbott and Jones 2015; Knoblauch et al. 2021; Marushchak et al. 2021; Melchert et al. 2022).

Incubation studies on permafrost‐derived DOC have shown the high lability of this carbon pool (e.g., Mann et al. 2014, 2015; Textor et al. 2019; Vonk, Mann, Davydov, et al. 2013) and DOC has been characterized as the labile carbon pool also in whole‐water incubation studies (Shakil et al. 2022; Tanski et al. 2019). This is supported by the modern age of DOC in fluvial networks in the Arctic suggesting that the old, permafrost‐derived DOC has already been mineralized (Dean et al. 2020; Drake et al. 2018; Rogers et al. 2021). However, better understanding the role of particulate OC (POC) in carbon dynamics (alongside DOC incubations) is essential, as abrupt permafrost sites release an order of magnitude more POC than DOC (e.g., Shakil et al. 2020). While POC might degrade and add to the GHG emissions, the presence of particles during incubations may also facilitate processes such as mineral‐OC interactions that affect OC availability to degradation, nutrient dynamics as well as processes such as weathering that are important to consider for the overall CO_2_ emission balance (Keskitalo et al. 2022; Melchert et al. 2022; Opfergelt 2020). To better understand in situ processes, incubation studies should include both dissolved and particulate matrices (following e.g., this study or Shakil et al. 2022).

**FIGURE 1:**
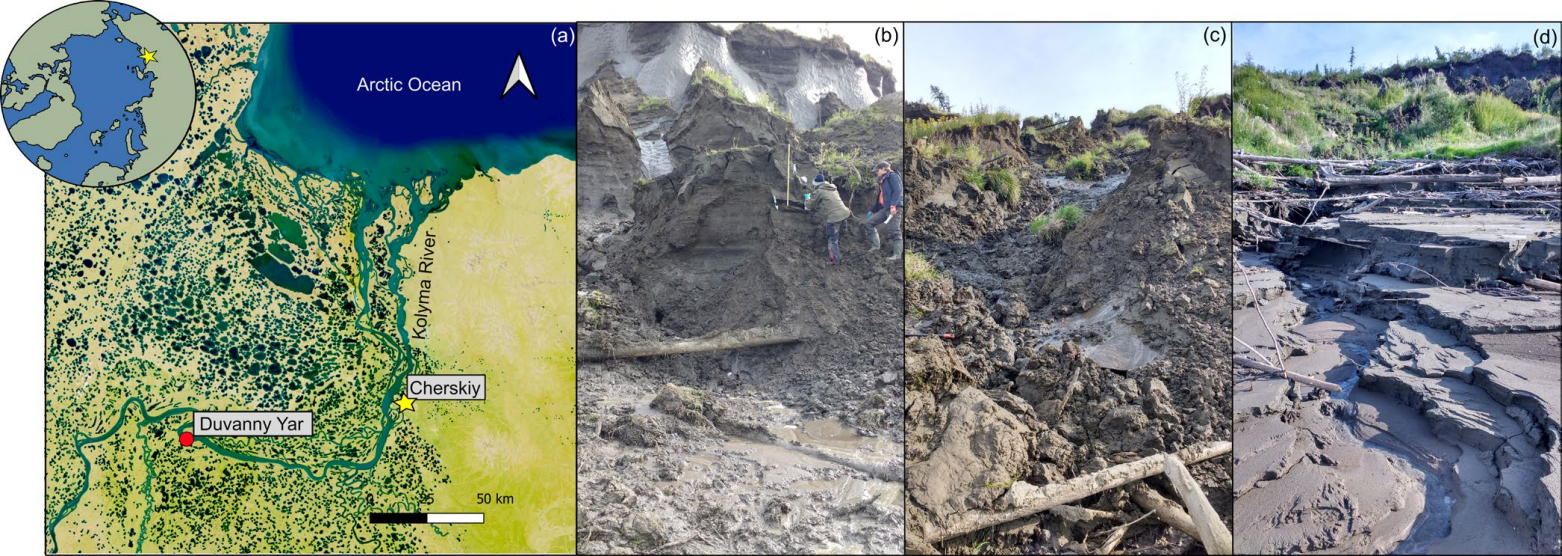
(a) The Duvanny Yar permafrost outcrop is located along the Kolyma River, upstream from the Northeast Science Station in Cherskiy. Background map is adapted from an ESRI satellite image. Map lines delineate study areas and do not necessarily depict accepted national boundaries. Sampling locations of thaw streams (b) DY1, (c) DY2, and (d) DY3. Samples were taken where the photographer stands.

Here, we incubated permafrost thaw stream waters (both as whole‐water and dissolved only fractions) at an abruptly thawing permafrost site known as ‘Duvanny Yar’ and additionally, waters at the outflow of thaw streams to the Kolyma River in northeastern Siberia (Figure 1a). Additionally, we incubated filtered waters to assess microbial degradation on the dissolved fraction (DOC only). All incubations were executed in the dark (to minimize biological production) and at room temperature (~15°C). We measured headspace gases (CO_2_, CH_4_, and N_2_O) and dissolved O_2_ and pH. Furthermore, we analysed DOC, POC and dissolved inorganic carbon (DIC) concentrations and their δ^13^C at three time points during the incubations to track changes (e.g., due to microbial degradation) in these different carbon pools. We also measured Δ^14^C‐POC for the outflow water incubations to see if there was any preferential degradation of younger/older carbon. Finally, we analysed δ^13^C‐OC, δ^13^C‐DIC, Δ^14^C‐POC, and δ^18^O of the thaw streams and fluvial outflow sites to characterize their composition. This study aims to address GHG emissions from permafrost thaw streams using a whole‐water incubation method mimicking in situ conditions (i.e., turbulent conditions in streams/rivers) combined with measurements of different carbon pools (DOC, POC, and DIC) with the objective to better couple vertical dynamics (GHG emissions) with lateral processes during downstream catchment transport. Furthermore, we aim to provide observational evidence for upscaling studies and to better understand the role of thaw streams in Arctic fluvial networks.

## 2 Materials and Methods

### 2.1 Study Area and Background

The entire Kolyma River watershed in northeastern Siberia is underlain by continuous permafrost (100% cover, Holmes et al. 2012). The abruptly thawing permafrost site called ‘Duvanny Yar’, is located ~210 km upstream from the Kolyma River mouth (N68.63042, E159.15475; Figure 1a). The outcrop releases permafrost carbon, predominantly in the form of POC, via thaw streams and slumping into the Kolyma River mainstem (Vonk, Mann, Davydov, et al. 2013). The permafrost at the site consists of late Pleistocene Yedoma Ice Complex and Alas deposits (Vasil'chuk and Vasil'chuk 1997). The Yedoma complex dates back to the Last Glacial period (Lindgren et al. 2016; Strauss et al. 2017) and is characterized by high OC (2%–5%) and ground ice content (up to 75% of volume; Strauss et al. 2012; Zimov et al. 2006). The sediments are poorly/very poorly sorted, dominantly silty sediments (Strauss et al. 2012) consisting mainly of quartz, feldspar plagioclase, micas and kaolinite (Monhonval et al. 2021). Previous studies at Duvanny Yar have focused on the dissolved carbon fractions and their biological and photochemical lability (e.g., Drake et al. 2018; Mann et al. 2015; Stubbins et al. 2016; Vonk, Mann, Davydov, et al. 2013), physical characteristics and sediment composition (e.g., Monhonval et al. 2021; Murton et al. 2015; Strauss et al. 2012), ice‐wedges (Vasil'chuk et al. 2001; Vasil'chuk and Vasil'chuk 1997), and paleoenvironmental reconstruction and depositional processes (Shmelev et al. 2021; Zanina et al. 2011). However, studies on incubation‐based GHG emissions using whole‐water samples from the thaw streams are lacking.

### 2.2 Field Sampling

At Duvanny Yar, thaw stream waters (extremely saturated with sediment) were collected from three locations (DY1, DY2, and DY3; Figure 1b) for the incubation experiments. DY1 was closest to the actively thawing headwall with large, massive ice wedges directly above. DY2 was further away from the headwall, while DY3 was sampled farthest downstream (~10 m from the headwall). Additionally, we collected waters from two outflow sites of thaw streams to the Kolyma River next to the riverbank.

All samples were collected in July/August 2018 from the surface of the thaw streams using a 1 L pre‐rinsed Nalgene bottle. The outflow samples were collected ~1 m distance from the riverbank, next to Duvanny Yar, at a depth of ~20 cm below the water surface. Surface waters were directly sampled for dissolved gas concentrations (CO_2_, CH_4_, and N_2_O) in the field using headspace equilibration (atmospheric air; a 50 mL syringe filled with 30 mL of water and 20 mL of air, shaken vigorously for 1 min and let to equilibrate) and collection (i.e., extracted with a needle) in pre‐evacuated 12 ml Exetainers (Labco, UK). Atmospheric air samples (at ~2 m height) were also collected on‐site for background air gas concentrations (*n* = 9). Additionally, we collected water samples for water isotope analysis (δ^18^O and δ^2^H).

All water samples were filter within 12 h of sampling using pre‐combusted (350°C, 6 h) glass‐fiber filters (Whatman, 0.7 μm). These served as the initial, non‐incubated time point (T_0_) for the incubations. Before filtering, samples were vigorously shaken to ensure particle mixing and resuspension. The filters were immediately frozen to −20°C, while the filtrate (~30 mL) was acidified with HCl (30 μL, 37%) and stored cool (+5°C). Samples for analyzing DIC were collected by injection of 4 mL of water, through in‐line syringe filters (Whatman, 0.7 μm) into a pre‐evacuated 12 mL Exetainer containing 50 μL of H_3_PO_4_. These samples were stored cool (+5°C) and dark until analysis. The samples for stable water isotopes (δ^18^O, δ^2^H) were filtered and stored cool (+5°C) without headspace. Additionally, for this study, we use previously published OC and DIC concentration and composition data from the Kolyma River mainstem for context (Keskitalo et al. 2022). All fieldwork was supported by the Northeast Science Station in Cherskiy.

### 2.3 Incubations

Water samples were prepared for incubation within 12 h of sampling. Waters were thoroughly shaken to ensure complete mixing and resuspension of particles and poured into 120 mL pre‐furnaced (350°C, 6 h) glass bottles (Wheaton) with three replicates for each sample. A 40 mL headspace was left in each bottle, except for two of the outflow sites (OF1 and OF2) that were incubated without headspace to be able to monitor dissolved O_2_ without interference from headspace O_2_. The samples were incubated using a custom‐made rotating device (see Keskitalo et al. 2022), which keeps particles in constant suspension mimicking stream/river conditions. The incubations were executed in dark conditions and at room temperature (15°C ± 1°C for thaw streams, 15°C ± 5°C for outflow sites). While we did not measure thaw stream water temperatures, our incubation temperatures likely reflect in situ water temperatures that can vary greatly as shown in runoff waters from retrogressive thaw slumps on the Peel Plateau in Canada (from 4.2°C to 19.3°C; Zolkos et al. 2019). Dissolved oxygen (DO) concentrations were monitored throughout the incubations, and incubations were stopped once DO reached 0. The thaw stream incubations lasted for 27–38 h before all the DO was depleted from the waters (note that headspace O_2_ was not measured). The fluvial outflow incubations lasted for 6 days and in contrast to the thaw streams, remained oxygenated until the end of the incubation.

At DY2 and DY3 thaw streams, replicate sample bottles (T_1_ *n* = 4 at DY2, *n* = 3 at DY3; T_2_ *n* = 2 at both DY2 and DY3; T_3_ *n* = 3 at DY2 and *n* = 4 at DY3) were removed at three set time‐points (17, 27, and 38 h), while at DY1, only at two time points (17 and 27 h) were measured due to quick oxygen loss (T_1_ *n* = 2; T_2_ *n* = 6). For fluvial outflow incubations, three set time points (*n* = 3 at each time point) were taken after 48 h, and then subsequently at 4 and 6 days. At each set time point, replicate bottles were removed from the rotator and the headspace sampled for GHG concentrations (CO_2_, CH_4_, and N_2_O; not measured for non‐headspace bottles). Subsequently, waters were filtered (as previously) for measurements of DIC, DOC, and POC fractions. Additionally, pH was measured at each timepoint using a hand‐held pH meter (Voltkraft PH‐410). Due to the high sediment load of the thaw stream waters, only subsamples (1–2 mL per sample, four replicates per bottle) from each bottle were filtered (to collect POC samples), while for the fluvial outflow incubations, all waters were filtered. We calculated GHG production as CO_2_ and CH_4_ per g dry weight (gdw) of sediment and as CO_2_‐C and CH_4_‐C gdw following Jongejans et al. (2021). Parallel to the whole‐water incubations, we incubated filtered waters (GFF Whatman, 0.7 μm) under identical conditions to quantify DOC degradation without particle presence. In brief, filtered waters (~30 mL) were incubated in 40 mL amber glass vials with loose lids to keep them oxygenated. The samples were shaken daily. The protocol is described in detail in Vonk et al. (2015). The thaw stream waters (both whole‐water and filtered incubations) visibly flocculated after the incubations. These samples were filtered to collect the flocculates which were subsequently analyzed for POC concentrations and δ^13^C‐POC.

### 2.4 Organic Carbon Analyses and Carbon Isotopes

The amount of total suspended solids (TSS, mg L^−1^) was calculated by the difference in dry filter weight before and after filtering, divided by the volume of water filtered.

For POC concentrations, δ^13^C‐POC, and total particulate nitrogen (TPN) filters were freeze‐dried and subsampled by punching approximately 18% of the 45 mm filter area and fitted into silver capsules/boats. The subsamples were treated with 1 M HCl to remove inorganic carbon and then placed into an oven at 60°C until dry. Afterwards, the samples were wrapped in tin capsules to aid combustion during analysis. The samples were analyzed at the Institute of Polar Sciences (CNR, Bologna, Italy) using a ThermoFischer DeltaQ coupled with a Flash2000 Elemental Analyzer.

For the ^14^C analysis, filters (see above for the subsampling method) were fumigated over 37% HCl (72 h at 60°C) to remove all inorganic carbon. After fumigation, samples were neutralized of excess acid (60°C, a minimum of 48 h) in the presence of NaOH pellets, and subsequently wrapped in tin boats. The samples were analyzed using a coupled elemental analyzer‐accelerator mass spectrometer (EA‐AMS) system (vario MICRO cube, Elementar; Mini Carbon Dating System MICADAS, Ionplus, Dietikon, Switzerland) (Synal et al. 2007). The filter samples were blank corrected for constant contamination according to the method presented in Haghipour et al. (2019). The ^14^C analysis was carried out at the Laboratory of Ion Beam Physics at the Swiss Federal Institute of Technology (ETH), Zürich, Switzerland.

The DOC samples from thaw streams were analyzed for OC concentrations and δ^13^C‐DOC at the North Carolina State University, Raleigh, USA. For the method details, see Osburn and St‐Jean (2007). The DOC samples from the outflow sites were analyzed for OC concentrations and δ^13^C‐DOC following a method described in Deirmendjian et al. (2020) at KU Leuven, Belgium. Flocculates in thaw stream DOC samples were analyzed for total organic carbon (TOC) concentrations, δ^13^C and total nitrogen (TN) concentrations at the Stable Isotope Facility (SIF) of the University of California, Davis, USA with an Elementar Vario EL Cube or Micro Cube elemental analyzer (Elementar Analysensysteme GmbH, Hanau, Germany) interfaced to a PDZ Europa 20–20 isotope ratio mass spectrometer (Sercon Ltd. Cheshire, UK) following their standard procedures. All OC data are available in Keskitalo et al. (2025).

### 2.5 Inorganic Carbon Analyses and Isotopes

For the DIC concentration and δ^13^C‐DIC analysis, headspace CO_2_ of the DIC samples were analyzed using a Gasbench interfaced to a Thermo Delta V IRMS at the Northumbria University, UK. Analytical standard deviation for the instrument was < 0.15‰. Dissolved, headspace, and atmospheric gases (CO_2_, CH_4_, and N_2_O) were measured using gas‐chromatography (Shimadzu GC‐2014) at the Woodwell Climate Research Center, USA following their standard procedures. All DIC data are available in Keskitalo et al. (2025).

### 2.6 Water Isotopes

Water stable isotopes (δ^18^O, δH) were analyzed using a cavity ring‐down spectrometer (Picarro Inc. L2140‐i, Vrije Universiteit Amsterdam, Netherlands) in seven measurement replicates, of which the first three were discarded to avoid carry‐over effects. After each 10 samples, three in‐house standards, calibrated against the international standards, VSLAP and VSMOW (provided by the IAEA), were analyzed. A fourth in‐house standard (KONA), was used to control the precision and accuracy of the measurements. The analytical standard deviation was < 0.1‰ for δ^18^O and < 2‰ for δH. Water isotope data are available in Keskitalo et al. (2025).

### 2.7 Degradation Constant

Degradation constant (*k*) was calculated for the DOC pool in both the whole‐water and filtered water incubations following Attermeyer et al. (2018) and Richardson et al. (2013). The degradation/loss rates were calculated using a first degree exponential model (Equation 1):

(1) $${\mathrm{OC}}_{t}={\mathrm{OC}}_{\text{init}}e^{-\mathrm{kt}}$$

where *k* is the degradation constant, OC_*t*_ is the residual OC concentration at the end of the incubation, OC_init_ is the initial OC concentration and *t* is the incubation time in days.

### 2.8 Statistical Analysis

We used a linear regression model (function *lm*) to test the linear relationship between DOC losses and gains in DIC. We assessed the normality, independence, and homoscedasticity of the residuals. To test whether there were significant differences in POC, DOC, DIC, δ^13^C‐OC, δ^13^C‐DIC, Δ^14^C, TSS, TPN, POC/TPN ratio, and δ^18^O between thaw streams and the outflow sites we used a Welch's *t*‐test. Prior to the test, we assessed the normality of the data using a Shapiro–Wilks test. For data that did not fulfil the assumptions of normality, we used a Mann–Whitney *U* test. Similarly, we used Welch's *t*‐test to assess whether there were significant differences in the abovementioned parameters between the Kolyma River thalweg and the outflow sites. Furthermore, we used Welch's *t*‐test to assess whether GHG emissions at the end of the incubation differed significantly from the initial conditions. Furthermore, an analysis of variance (ANOVA) or Kruskal–Wallis test was used to examine if gains/losses of TSS, POC, DOC, δ^13^C‐OC, TPN, and POC/TPN were significantly different from the initial (T_0_) combined with a Tukey's test or a Dunn's test as post hoc tests. All statistical testing was conducted in R (R Core Team 2020).

## 3 Results

### 3.1 Initial Conditions in Thaw Streams and Fluvial Outflow Sites

**FIGURE 2:**
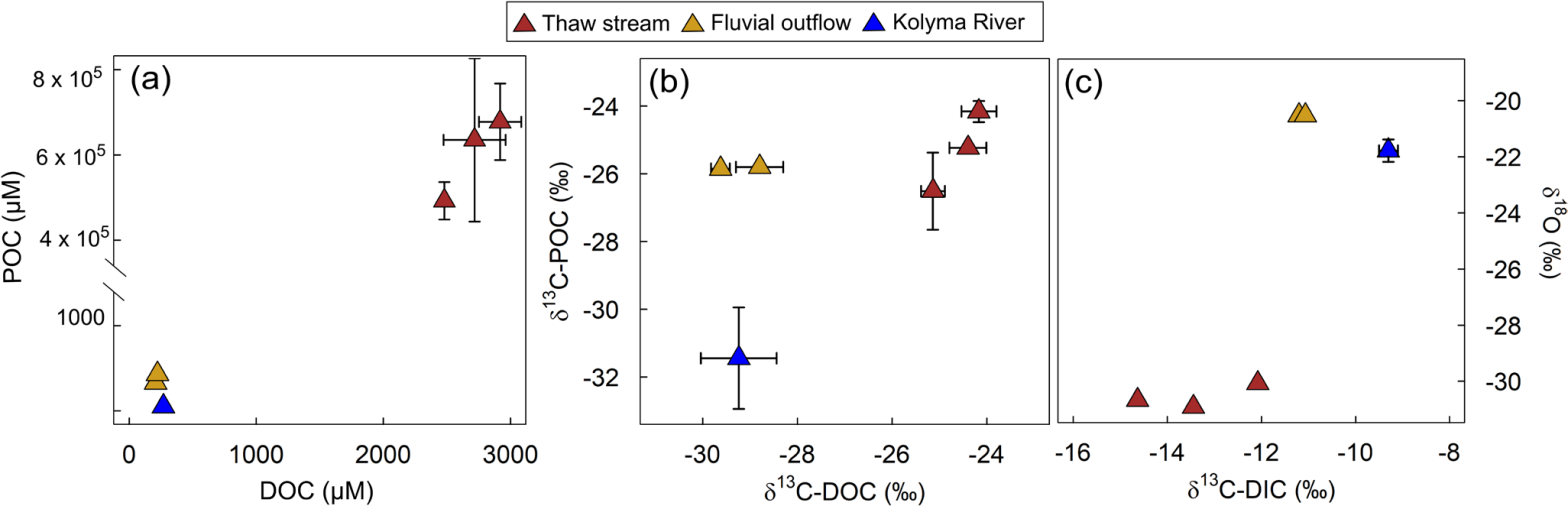
Initial conditions in permafrost thaw streams (red triangles), fluvial outflow sites (yellow triangles) and for comparison Kolyma River thalweg (blue triangle). (a) Particulate and dissolved organic carbon (POC and DOC, respectively) concentrations. (b) The δ^13^C of POC and DOC. (c) The δ^13^C of dissolved inorganic carbon (DIC) and δ^18^O. Mean and standard deviation between replicate samples is shown in all panels (standard deviation not shown if smaller than the symbol). For panel (c), there were no replicates except for the Kolyma River mainstem. Kolyma River mainstem data have been previously reported in Keskitalo et al. (2022).

Water oxygen isotopes (δ^18^O) were significantly depleted (−30.55‰ ± 0.43‰) in the thaw streams relative to those in fluvial outflow sites (−20.57‰ ± 0.09‰, *p* < 0.001; Figure 2; Table 1; Table S1). Thaw streams contained high dissolved CO_2_ (2332 ± 138 ppm; Table S2), and were supersaturated along with N_2_O concentrations (0.59 ± 0.3 ppm) relative to the measured atmospheric GHG concentrations (CO_2_ = 413 ± 6.4 ppm, CH_4_ = 1.6 ± 0.2 ppm, N_2_O = 0.24 ± 0.02 ppm, *n* = 9; Table S2). Dissolved CH_4_ concentrations (2.71 ± 0.7 ppm) were supersaturated at all thaw streams. In situ dissolved GHG concentration data are not available for the fluvial outflow sites.

**TABLE 1:** Initial conditions in permafrost thaw streams (DY1, DY2, DY3), fluvial outflows (OF1, OF2) and Kolyma River mainstem including concentrations of particulate and dissolved organic carbon (POC and DOC, respectively), dissolved inorganic carbon (DIC), ratio between POC and total particulate nitrogen (TPN), stable carbon isotopes (δ^13^C) of all the carbon pools and water isotopes (δ^18^O). Kolyma data are reported previously in Keskitalo et al. (2022).

Site	TSS (g L^−1^)	POC (mM)	POC‐%	POC/TPN	Δ^14^C‐POC (‰)	δ^13^C‐POC (‰)	DOC (μM)	δ^13^C‐DOC (‰)	DIC (μM)	δ^13^C‐DIC (‰)	δ^18^O (‰)
DY1	595 ± 304	626 ± 199	1.2	9.4 ± 0.6	−833	−26.51 ± 1.14	2705 ± 254	−25.14 ± 0.2	3695 ± 1.2	−12.08 ± 0.09	−30.07 ± 0.18
DY2	812 ± 121	570 ± 11	0.7	8.8 ± 0.1	−861	−25.23 ± 0.2	1968 ± 46	−24.40 ± 0.4	4427 ± 2	−14.63 ± 0.01	−30.67 ± 0.07
DY3	850 ± 53	670 ± 94	0.9	8.7 ± 0.3	−856	−24.16 ± 0.3	2164 ± 149	−24.17 ± 0.4	2769 ± 1.6	−13.44 ± 0.03	−30.92 ± 0.04
OF1	0.218 ± 0.01	431 ± 9 (μM)	2.4 ± 0.1	9.2 ± 0.3	−853 ± 8	−25.80 ± 0.1	223 ± 5.4	−28.80 ± 0.5	525 ± 0.2	−11.20 ± 0.03	−20.61 ± 0.10
OF2	0.189 ± 0.02	330 ± 23 (μM)	2.1 ± 0.1	9.1 ± 0.1	−860	−25.85 ± 0.1	208 ± 14	−29.62 ± 0.2	468 ± 0.1	−11.06 ± 0.05	−20.52 ± 0.02
Kolyma	0.015 ± 0.01	51.7 ± 13 (μM)	4.2 ± 0.9	7.6 ± 0.9	−273 ± 77	−31.44 ± 1.5	271 ± 26	−29.24 ± 0.8	473 ± 56	−9.30 ± 0.2	−21.78 ± 0.40

Thaw stream POC concentrations (up to 670,000 μM) were significantly higher than those in the fluvial outflow sites (330–431 μM, *p* < 0.001; Figure 2a; Table 1; Table S1). Similarly, DOC and DIC concentrations were significantly higher in thaw streams (1968–2705 μM and 2769–4427 μM, respectively) as compared to fluvial outflow waters (208–223 μM and 468–252 μM, respectively, *p* < 0.001 for DOC, too few data to test statistical significance of DIC; Table 1; Table S1).

The Δ^14^C‐POC signature did not significantly differ between the thaw streams (−850‰ ± 15‰, corresponding to 15,200 years uncalibrated radiocarbon ages) and the fluvial outflow sites (−853‰ ± 8‰, 15,400 years in uncalibrated radiocarbon ages, *p* > 0.05; Table 1; Table S1). The δ^13^C‐POC in the thaw streams and fluvial outflow sites were in a similar range (−25.30 ± 1.2 and −25.82‰ ± 0.04‰, respectively, *p* > 0.05, Figure 2b; Table 1; Table S1). In contrast, the δ^13^C‐DOC signature at fluvial outflow sites (−29.21 ± 0.6) was significantly lower than in the thaw streams (−24.57‰ ± 0.5‰, *p* < 0.001; Figure 2b; Table 1; Table S1). For δ^13^C‐DIC, thaw streams showed lower values, ranging from −14.63‰ to −12.08‰, while the δ^13^C‐DIC signature was higher in the fluvial outflow waters (−11.12‰ ± 0.1‰; Figure 2c; Table 1).

### 3.2 Carbon and Nitrogen Concentrations and Carbon Isotopes During Incubations

#### 3.2.1 Particulate Carbon and Nitrogen Concentrations and Carbon Isotopes

During the whole‐water incubations, the amount of POC increased in thaw stream samples by between 5% and 45% over 38 h (significant only at DY2), while TSS either decreased by −1.6% and −22% (DY2 and DY3, respectively) or increased by 41% (DY1; Table 2; Figure S1a). Concentrations of TPN increased by 14% at DY1 (7800 μM, p > 0.05) and by 36% (16,700 μM, *p* < 0.01) at DY2 while they decreased by −0.4% (−250 μM, p > 0.05) at DY3.

**TABLE 2:** Changes in particulate and dissolved organic carbon (POC and DOC, respectively), dissolved inorganic carbon (DIC) and carbon isotopes (δ^13^C) of all carbon pool after whole‐water incubations at permafrost thaw streams (DY1, DY2, DY3) and fluvial outflow sites (OF1HS, OF1, OF2). Also shown are changes in DOC in filtered (DOC only) incubations. The % here refers to gain in POC concentrations in percentage. Note that n/a refers to ‘not applicable’. Flocculation is not accounted for in this table. For the flocculation data, see Table S2.

					POC			Whole‐water DOC			DIC			Separate DOC		
Site	Head‐space	Hours (days)	TSS (g L^−1^)	TPN (μM)	μM	%	δ^13^C (‰)	μM	%	δ^13^C (‰)	μM	%	δ^13^C (‰)	μM	%	δ^13^C (‰)
DY1	Yes	27 (~1.1)	+246	+7840	+49,200 ± 227,300	+8	+0.68	−692 ± 281	−34	+0.36	+12,100 ± 1900	+327	−6.85	−217 ± 315	−11	+1.15
DY2	Yes	38 (~1.6)	−13	+16,690	+215,100 ± 120,500	+45	−0.30	−790 ± 146	−40	−0.05	+9300 ± 1800	+210	−3.87	−102 ± 175	−5	+0.85
DY3	Yes	38 (~1.6)	−200	−245	+32,200 ± 159,100	+5	+0.04	−530 ± 252	−27	+0.74	+5200 ± 4600	+90	−4.42	+111 ± 235	+5	+0.78
OF1HS	Yes	144 (6)	+0.048	+11	+94 ± 23	+22	−0.19	−22 ± 6	−10	+0.47	+70 ± 42	+13	−0.99	−37 ± 17	−17	−0.34
OF1	No	144 (6)	+0.043	+10	+129 ± 15	+30	−0.34	−22 ± 8	−7.2	+0.26	+57 ± 37	+11	−0.94	n/a	n/a	n/a
OF2	No	144 (6)	+0.047	+3.2	+67 ± 73	+20	−0.09	−16 ± 22	−7.6	+1.16	+59 ± 19	+12	−1.09	−23 ± 37	−11	+0.61

Abbreviations: DOC, dissolved organic carbon; OF, out flow; POC, particulate organic carbon; TPN, total particulate nitrogen; TSS, total suspended solids.

In the fluvial outflow waters, concentrations of POC at site OF1 and OF1HS significantly increased by +22% to 30% (+94 to 129 μM, *p* < 0.001) during 6 days of incubation, while TSS increases (+20% to 25%, +43 to 48 mg L^−1^) were significant only at OF1HS and OF2 (*p* < 0.04; Table 2; Table S3; Figure S1b). Similarly, TPN increases of +10% to 27% (+3 to 11 μM) were significant at OF1HS (*p* < 0.001; Table S3).

During incubation of both thaw stream and outflow waters, the δ^13^C‐POC did not show significant changes between the initial and last time point (Table 2; Tables S3 and S4). The Δ^14^C‐POC during the outflow incubations showed no significant change (only measured for outflow sites; Tables S4 and S5).

#### 3.2.2 Dissolved Carbon Concentration and Carbon Isotopes

During incubations of the thaw stream waters, concentrations of DOC decreased by −24% to 40% (−530 to 790 μM, Figure S1a, *p* < 0.05 at DY1 and DY3), while those of DIC increased by +90% to 210% (+5198 to 9281 μM). The thaw stream DOC visibly flocculated in the vials afterwards and the formed flocs contained an extra 24% to 39% of DOC (effectively transformed to POC; Table S6). The trends in DOC losses during incubations remained the same even when the flocs were added back to the DOC pool (Figure S2a). Fluvial outflow waters showed a similar trend with DOC decreasing by −7% to10% (*p* < 0.05 at OF1 and OF1HS) and DIC increasing by 11%–13% (Table 2; Table S4) (no flocculation was observed for these samples). The degradation constant (*k*, calculated only for the DOC pool of the incubations) in thaw stream waters was higher (−0.290 ± 0.100 day^−1^, *n* = 3) than in the outflow sites (−0.014 ± 0.003 day^−1^, *n* = 3; Table S7). A linear regression model shows that DOC losses explain 96.6% of the DIC gains (Figure S4).

The thaw stream δ^13^C‐DOC decreased at DY2 (−0.05‰) during incubations, while they increased at DY1 and DY3 (+0.36‰ and +0.74‰, respectively; Table 2; Table S3). The δ^13^C‐DIC decreased during thaw stream incubations between −6.85‰ and −3.87‰. In the fluvial outflow waters, the δ^13^C‐DOC increased by +0.26‰ to 1.16‰ while the ^13^C signature of DIC decreased by −0.94‰ to 1.09‰ (Table 3; Table S4).

**TABLE 3:** Changes in headspace gases (CO_2_, CH_4_, N_2_O) during whole‐water incubations at permafrost thaw streams (DY1, DY2, DY3) and incubation of fluvial outflow waters (OF1HS). Samples OF1 and OF2 were incubated without headspace.

Site	Hours (days)	CO_2_ (ppm)	CO_2_ (%)	CH_4_ (ppm)	CH_4_ (%)	N_2_O (ppm)	N_2_O (%)
DY1	27 (~1,1)	+34,960 ± 1660	+8620	+8.8 ± 0.8	+471	−0.18 ± 0.03	−85
DY2	38 (~1.6)	+20,780 ± 7760	+5080	+5.3 ± 4.2	+250	−0.15 ± 0.04	−62
DY3	38 (~1.6)	+18,030 ± 9870	+4450	+3.0 ± 1.6	+89	+0.57 ± 1.1	+224
OF1HS	144 (6)	+332 ± 36	+180	+5.9 ± 0.4	+281	+0.03 ± 0.02	+12
OF1	144 (6)	n/a	n/a	n/a	n/a	n/a	n/a
OF2	144 (6)	n/a	n/a	n/a	n/a	n/a	n/a

Abbreviation: OF, out flow.

During separate filtered incubations (i.e., without particles), DOC concentrations decreased by −11% (−217 ± 315 μM) at DY1 and by −5.2% (−102 ± 175 μM) at DY2, while they increased by 5.1% (111 ± 235 μM) at DY3 (Figure S2b; Tables S6 and S8). Similar to the whole‐water incubations, flocculation occurred in the vials during incubation amounting to overall increases in DOC (in the form of flocs) ranging between +220 μM and +390 μM at the end of the incubation (Figure S2b; Table S6). In the outflow waters, DOC showed losses between −11% and −17% (−23 ± 37 μM and −37 ± 17 μM) without any flocculation (Table S6). The degradation constant showed the same trend as in the DOC pool of the whole‐water incubations: it was higher in the thaw stream filtered waters (−0.067 ± 0.047, *n* = 2) than those of the outflow sites (−0.030, *n* = 1; Table S7).

### 3.3 Changes in Dissolved Oxygen and Greenhouse Gases (CO_2_ , CH_2_ , N_2_O) During Incubations

Initial (T_0_) DO concentrations in thaw streams were lower (4.5 ± 0.6 mg L^−1^) than those in the fluvial outflow (7.2 ± 0.4 mg L^−1^, *p* < 0.001; Table S1). The DO concentrations in thaw streams dropped 55%–75% within 18 h and were fully or nearly fully depleted within 36 h (< 1.2 mg L^−1^). Fluvial outflow sites remained oxygenated (O_2_ saturation ≥ 70%) until the end of all incubations (for 6 days; Table 2). Water DO saturation (%), accounting for any temperature or pressure variations, decreased exponentially during all the incubations (Figure S3).

Headspace CO_2_ significantly increased by 18,000 to 35,000 ppm (4500%–8600%, *p* < 0.05) during thaw stream incubations while in the fluvial outflow, the increase was 332 ppm (180%, *p* < 0.001; Table 3; Table S9). The CH_4_ increased by 8.8 ± 0.8 ppm (471%, *p* < 0.001) in thaw stream DY1, while the in the other thaw streams the increases were insignificant (Table 3; Table S9). In the fluvial outflow site (OF1HS) CH_4_ concentrations significantly increased by 4.3 ppm (280%; *p* < 0.001; Table 3; Table S9). For both CO_2_ and CH_4_, increases were highest for DY1, decreasing with increasing distance from the headwall. The N_2_O concentration decreased by −0.18 (−85%) and by −0.15 ppm (−62%) in thaw streams DY1 and DY2 (*p* < 0.001), respectively (Table 3; Table S9). In thaw stream DY3 and in the fluvial outflow site OF1HS, the increases in N_2_O were insignificant (Table 3; Table S9).

The increase in CO_2_ per gram dry weight (gdw) of suspended sediment was 11.7–33.7 μg CO_2_ gdw^−1^ day^−1^ for thaw stream waters, while for the fluvial outflow site it was 533 ± 18 μg CO_2_ gdw^−1^ day^−1^ (Figure 4; Table S10). For CH_4_, the increase was 0.001–0.003 μg CH_4_ gdw^−1^ day^−1^ in thaw streams and 8.2 ± 0.5 μg CH_4_ gdw^−1^ day^−1^ in the outflow site (Figure 4; Table S10).

## 4 Discussion

### 4.1 Thaw Stream Carbon Dynamics During Mobilization

Thaw streams were characterized by extremely high TSS (> 800 g L^−1^), POC (up to 670,000 μM), and DOC (> 1900 μM) concentrations, in agreement with previous studies (e.g., Vonk, Mann, Davydov, et al. 2013). Thaw stream waters were formed primarily from ice‐wedge melt waters as determined by isotopic signatures (Vonk, Mann, Dowdy, et al. 2013) which subsequently mobilizes very old POC (Δ^14^C ~900‰, corresponding to an uncalibrated radiocarbon age of about 15,000 years) rich in inorganic carbon (i.e., low POC‐%). Thaw streams were supersaturated in dissolved CO_2_ (1993 ± 120 ppm), and in their CH_4_ concentrations (2.71 ± 0.7 ppm, Table S2). These high concentrations might be due to high reactivity of old OC and preferential loss of Yedoma carbon within thaw streams immediately upon thaw (Mann et al. 2015).

In the outflow waters, TSS and POC concentrations were significantly lower (189–218 mg L^−1^ and 330–431 μM, respectively; Table 1; Table S1) than those in thaw streams, likely due to rapid settling of particles to the riverbed (Jong et al. 2023). Despite this, suspended sediments were still dominated by old, thaw stream material (Δ^14^C ~900‰), with thaw stream sediments dominating the POC pool (~85%; Table 1). Particle composition (δ^13^C‐POC, POC/TPN) resembled those of thaw streams (Table 1; Table S1) suggesting that riverbank particle load mirrors Yedoma deposits they originate from. However, the POC‐% was higher (> 2%) in the outflow waters likely due to autochthonous production contributing to the Kolyma River POC pool (Table S1; Jong et al. 2023; Keskitalo et al. 2022), contribution of contemporary biomass (Wild et al. 2019 and references herein) and/or selective settling of older POC. Kolyma River POC (sampled from the thalweg; data from Keskitalo et al. 2022) consists of more autochthonous (δ^13^C‐POC −31.44 ± 1.5) and younger organic matter (Δ^14^C −273 ± 77; Table 1).

The order of magnitude lower DOC and DIC concentrations in fluvial outflow waters (~200 and ~500 μM, respectively) likely represent rapid mixing and dilution with Kolyma River waters (Table 1). The composition of the dissolved fraction (δ^18^O, δ^13^C‐DOC) differed significantly (δ^18^O, δ^13^C‐DOC) between fluvial outflow and thaw stream waters (Table S1), the outflow resembling more closely waters from the Kolyma River thalweg than thaw streams.

### 4.2 Degradation Processes and Greenhouse Gas Emissions During Incubations

#### 4.2.1 Losses in Dissolved Organic Carbon Suggest Microbial Degradation

During whole‐water incubations, losses of DOC indicate microbial degradation of organic matter. This is supported by increases in DIC concentrations and decrease in δ^13^C‐DIC (Table 1) suggesting microbial degradation as shown in previous studies (e.g., Drake et al. 2018). Furthermore, a linear regression model shows that DOC losses explain 96.6% of the DIC gains, suggesting that DOC is re‐mineralized (Figure S4). The POC fraction shows gains suggesting that mostly DOC is degraded in the whole‐water incubations as proposed previously by Shakil et al. (2022) and Tanski et al. (2019). Yet roughly three to five times higher losses of DOC in whole‐water incubations vs. filtered incubations for the thaw streams suggest that DOC is not utilized solely in microbial respiration: up to a third of the DOC pool may interact with particles (Table S6). This is reflected in the degradation (or loss as flocculation) constants (*k*): whole‐water incubations show over four times higher *k*‐values, likely reflecting these interactions, than the filtered incubations (Table S7).

Our data show that thaw stream DOC is prone to flocculation as 24.8% ± 3.1% of DOC flocculated after the whole‐water incubations (similarly 31.6% ± 7.6% of DOC flocculated during/after the filtered incubations; Figure S2; Table S6). While with our data it is not possible to decipher how much flocculation might have happened already during the whole‐water incubations (as we only know how much happened after filtering the samples at each time point), the gain in POC‐% in DY2 and DY3 suggest that non‐mineral organic matter increases during incubations that could be attributed to flocculation (in contrast to DY1 where POC‐% decreases). The δ^13^C signature of the residual DOC versus flocs suggest that some fractionation of δ^13^C occurred already during the whole‐water incubations (i.e., prior to filtering): in the filtered incubations the δ^13^C signature showed larger fractionation between DOC and flocs (1.5‰ ± 0.6‰) compared to the DOC from the whole‐water incubations (0.8‰ ± 0.6‰), suggesting that DOC from the whole‐water incubations could already have fractionated during the incubations, thereby increasing the δ^13^C values of the residual DOC pool. While there are no studies showing this directly for flocculation, previous studies have shown that the preferential adsorption of hydrophobic compounds (with generally lower δ^13^C signature) to minerals can raise the δ^13^C values of the residual DOC pool (Kaiser et al. 2001). However, the relatively high pH (8.03–8.16, Table S11) and high TSS (Table 1) of the thaw streams investigated here may have reduced the DOC adsorption potential (Groeneveld et al. 2020). Similar to adsorption, our filtered incubations suggest that flocculation increases the δ^13^C signature of the residual DOC pool likely due to preferential flocculation of compounds with low δ^13^C.

In the fluvial outflow sites, the pattern between whole‐water and filtered incubations is the opposite to the thaw streams: DOC losses are higher in the filtered incubations than in the whole‐water incubations (Table 2). These trends are reflected in the degradation constants (*k*), where whole‐water incubations show two times slower degradation/losses than filtered incubations (Table S7). A similar pattern to the outflow waters was observed in the Kolyma River waters (sampled at thalweg) and attributed to leaching of POC adding DOC to the dissolved pool (Keskitalo et al. 2022). However, degradation constants in the Kolyma waters were lower (−0.006 ± 0.002 day^−1^, *n* = 3 in the whole‐water and −0.012 ± 0.002 day^−1^, *n* = 2 in the filtered waters) than in the outflow waters, suggesting that thaw streams likely provide labile organic matter to the outflow waters where it is rapidly utilized or interacts with particles. Additionally, leaching of POC may be more pronounced in the Kolyma River waters ‘slowing down’ the apparent degradation of DOC.

#### 4.2.2 Abrupt Permafrost Thaw Causes CO_2_ Release During Downstream Transport

**FIGURE 3:**
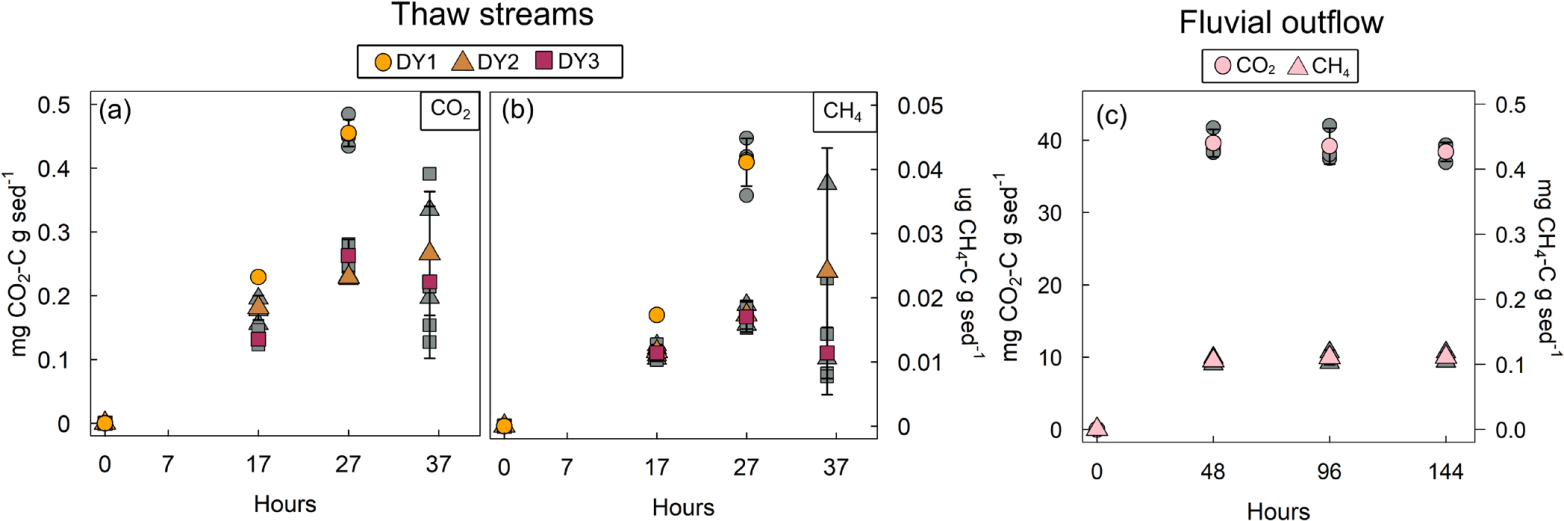
Increases in greenhouse gas (GHG) concentrations (CO_2_‐C and CH_4_‐C per g sediment dry weight^−1^, mean ± std) in the headspace during incubation of thaw stream waters (a, b) and fluvial outflow site (c). Individual measurements are shown in grey symbols. Note that *y*‐axes are in a different scale.

During thaw stream incubations, the up to 85‐fold increase in headspace CO_2_ (+18,000 to 35,000 ppm, +4500% to 8600%) suggests that Duvanny Yar may be a potential point‐source of GHG emissions (Table 3). The increase in CO_2_ concentrations is highest at DY1 (closest to the headwall) and decreases gradually for DY2 and DY3 farther away from the headwall. This suggests that the highest CO_2_ emissions occur in the beginning of the thaw streams closest to the thawing permafrost source and decrease farther downstream presumably after the most labile organic matter fractions have been utilized. Additionally, ice wedge melt waters have been shown to promote degradation (Vonk, Mann, Dowdy, et al. 2013) potentially contributing to higher CO_2_ emission closest to the headwall. In the fluvial outflow waters, the headspace CO_2_ increase was lower (+87%, +288 ppm) than those of thaw streams and plateau after 2 days. While one has to remain careful to derive conclusions from experimental incubations, this may suggest that the most labile components can be respired in the order of a few days (Figure 3; Table 3). In the thaw stream waters, the emissions start to plateau for DY2 and DY3 after 27 h, while for DY1 they increase linearly up to the same point (also the end point of that incubation; Figure 3; Table 3) implying fast degradation of the most labile pool. It is likely that not all labile compounds have degraded (aerobically) during our relatively short incubation. Tanski et al. (2019) show that initial, high turn‐over rates last for ~10 days, followed by slower turn‐over rates for 30 days before plateauing during their 3‐month 16°C incubation of coastal permafrost. Turn‐over times of the labile pool at colder temperatures (4°C) will be even longer (~95 days; Knoblauch et al. 2013).

**FIGURE 4:**
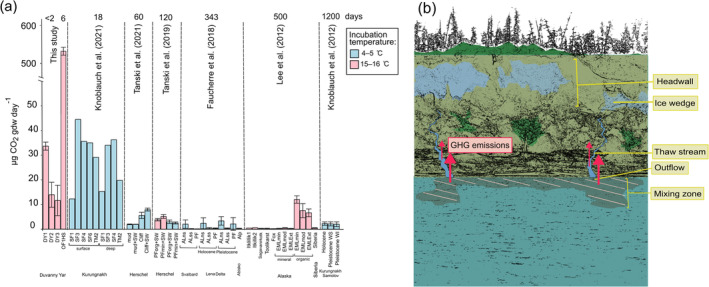
(a) Comparison of CO_2_ emissions from different incubation studies. In this study sediments were incubated in fresh water. In both Tanski et al. (2019, 2021) seawater was used for incubations. While in Tanski et al. (2019) sediment (mud, mud+SW) was incubated, in all the other studies soil samples were incubated in original soil moisture. Incubation days are shown above the figure. Standard deviations are given when available. AL, active layer; Alp, alpine; EML, Eight Mile Lake (min, minimal; mod, modest; ext., extensive refer to the extent of permafrost thaw); min, mineral; ns, near surface; org, organic; PF, permafrost; SF, slump floor; ss, subsurface; SW, seawater; TM, thaw mound; WI, Weichselian interstadial; WS, Weichselian stadial. See more details about the different incubation studies in Table S12. (b) Conceptual figure of riverbank erosion with fluvial release via thaw streams, stream outflows, and mixing zones in the main stem. The arrows indicate greenhouse gas (GHG) emissions from thaw streams and outflow sites, where the size of the arrows illustrates roughly the size of the emission normalized to sediment. The dashed line highlights the mixing zone. The figure is based on a photograph by Sandra Raab.

While the absolute CO_2_ emissions are higher from the thaw streams than from the fluvial outflow waters, the gains in CO_2_ per gdw^−1^ sediment day^−1^ are lower in the thaw streams (11.7–33.7 μg CO_2_ gdw^−1^ day^−1^) than in the fluvial outflow waters (533 μg CO_2_ gdw^−1^ day^−1^) that hold orders of magnitude less sediment than the thaw streams (Figure 4; Table S12). The CO_2_ emissions from thaw stream waters were on a similar order of magnitude as reported previously for permafrost mud lobes (within a thaw slump) and eroding coastal cliffs on the Canadian coast (1.94–7.91 μg CO_2_ gdw^−1^ day^−1^; Tanski et al. 2021) and for slump floor and thaw mounds at Kurungnakh Island at the Lena River Delta (average 32.12 μg CO_2_ gdw^−1^ day^−1^; Knoblauch et al. 2021; Figure 4; Table S12). In contrast, Faucherre et al. (2018) have reported lower CO_2_ emissions during an incubation study using samples from a Holocene terrace (0.83–1.99 μg CO_2_ gdw^−1^ day^−1^) and from an Ice Complex Deposit (0.53–2.14 μg CO_2_ gdw^−1^ day^−1^) at the Lena River Delta. However, these comparisons are not straightforward as our incubations were short and executed at a room temperature (< 2 days at 15°C) compared to those of Tanski et al. (2021) that lasted for 60 days at 4°C (also using seawater as an incubation medium), those of Knoblauch et al. (2021) that lasted for 18 days at 4°C and those of Faucherre et al. (2018) that lasted for 343 days at 4°C. Furthermore, soil samples in the studies by Faucherre et al. (2018) and Knoblauch et al. (2021) were incubated at in situ soil moisture content differing from our incubations of stream/river water. Another incubation study by Tanski et al. (2019) of mineral permafrost in seawater was conducted at a similar temperature to our incubations (16°C). Their results showed two to thriteen times lower CO_2_ release (2.5–5.13 μg CO_2_ gdw^−1^ day^−1^; Figure 4; Table S12) compared to our thaw stream incubations. This discrepancy could be due to the short time of our incubations (stopped as soon as O_2_ ran out vs. keeping them oxygenated for 120 days in Tanski et al. 2019) that capture the initial high emissions from the degradation of the most labile fraction. Additionally, the increased salinity of both incubations by Tanski et al. (2019, 2021) may affect degradation rates as OC degradation may accelerate or decelerate in seawater depending on the composition of the organic matter (Tanski et al. 2021). The CO_2_ release from the outflow waters was higher than in any of the other studies (Table S12) likely due to our different incubation set‐up with very low sediment amount (< 1 g) compared to other incubation studies that tend to use 3–100 g of sediment (Table S12) and our relatively short incubation period of 6 days.

While our and the abovementioned incubation studies aim to address microbial degradation of organic matter, in the whole‐water incubations (where particles are present), geogenic processes such as chemical weathering may contribute to CO_2_ emissions in addition to biological processes (e.g., Zolkos et al. 2018; Melchert et al. 2022). Melchert et al. (2022) have estimated weathering to contribute ~18% to total CO_2_ emissions from Yedoma Ice Complex at an abrupt permafrost thaw site at the Lena River. The composition of the Siberian Yedoma Ice Complex deposits (mainly quartz, feldspar plagioclase, micas and kaolinite) suggests that some of it might be easily weatherable such as feldspar plagioclase, dolomite and calcite (Monhonval et al. 2021). Weathering of sulfur‐bearing minerals such as pyrite to sulfuric acid has been shown to cause weathering of carbonates and subsequent CO_2_ emissions on the Canadian Peel Plateau (Zolkos et al. 2018), however, these minerals have not been detected in Duvanny Yar (Monhonval et al. 2021). With our incubation set‐up, we were not directly able to measure the contribution of weathering to the total CO_2_ emissions, but it is highly likely that part of the CO_2_ originates from geogenic processes given that CO_2_ release is much higher than what would be expected purely based on losses of O_2_ (Tables 2 and 3).

#### 4.2.3 Thaw Streams and Outflow Waters Can Be a Source of CH_4_

During the whole‐water incubations, headspace methane concentrations increase in thaw streams following the same trend as CO_2_ with the highest emissions from DY1 followed by DY2 and DY3 (Figure 3; Table 3). In the fluvial outflow site (OF1HS), absolute CH_4_ increases are higher than in thaw streams (Figure 3; Table 3). Part of the headspace CH_4_ may originate from the dissolved phase (initial dissolved CH_4_ concentration were 2.71 ± 0.7 ppm in thaw streams, Table S2). However, the higher emissions than the initial concentrations in the dissolved phase suggest additional production of CH_4_ . However, for DY1 and DY2, the emissions were higher than the initial concentrations in the dissolved phase, suggesting additional production of CH_4_. Production of CH_4_ in freshwater systems under aerobic conditions has been suggested to result from different processes (e.g., breakdown of methylated compounds, methanogenesis in anoxic microsites in particles) (Perez‐Coronel and Michael Beman 2022). While our data suggest that aerobic production of CH_4_ occurs both in thaw streams and fluvial outflow waters, it is not possible to decipher the exact pathway of the production. The thaw stream CH_4_ release (1–3 ng CH_4_ gdw day^−1^) compares well with those measured by Tanski et al. (2019) on mineral and organic‐rich permafrost (120 days, at 4°C and 16°C, incubated in seawater) with CH_4_ emissions of 0.1–4.6 ng CH_4_ gdw day^−1^ where highest emissions were observed for the organic‐rich permafrost. While their incubations lasted for 120 days, the CH_4_ emissions plateaued already after the first ~5 days.

### 4.3 Complexity of Including Particles in Incubations

During the whole‐water incubations, all sites showed gains in POC from 5% up to 45%. Previous whole‐water incubation studies have similarly reported increases in POC (Attermeyer et al. 2018; Keskitalo et al. 2022; Shakil et al. 2022; Tanski et al. 2019) that were attributed to DOC adsorption and/or flocculation and/or chemoautotrophic carbon sequestration. Shakil et al. (2022) suggested chemotrophic processes such as nitrification and oxidation of sulfur that sequester organic carbon (and consume O_2_) as an explanation to gains in POC. However, contributions of these processes in their similarly high‐sediment load incubations could only explain a fraction of the gains in POC that they observed. Other processes that consume O_2_ such as oxidation of iron and manganese may also occur, although their contribution has been shown to be minimal (Spieckermann et al. 2022). Additionally, primary production during the incubation or bacterial biomass growth could explain gains in POC. In this respect, we tried to minimize any biological growth by keeping incubations in the dark, and bacterial biomass has been shown to be a negligible source in lake water incubations (von Wachenfeldt et al. 2009). Gains in POC have been shown to be more prominent in mineral‐rich particle incubations compared to incubations rich in autochthonous POC or organic matter in general (Keskitalo et al. 2022; Tanski et al. 2019).

We conclude that presence of particles during incubations is crucial to better understand in situ processes in these thaw streams, but it poses challenges in disentangling the complexity of interactions (e.g., adsorption, flocculation, leaching) and various biogeochemical processes (e.g., microbial degradation, bacterial growth, weathering, oxidation processes such as nitrification and formation of sulfur) that may occur during incubations.

### 4.4 Riverbank Environments as Hotspots for Carbon Breakdown

Nearshore riverbank waters are the primary receptors of terrestrial carbon; however, river studies often exclude these areas when estimating river wide dynamics. While river discharge estimates commonly use width and depth integrated measurements, for constituents estimates these methods are less commonly applied. Our study shows that these environments significantly differ in their biogeochemistry from the thalweg (particle phase) and from thaw streams (dissolved phase; Tables S1 and S13). Additionally, microbial degradation of DOC is faster in these environments than in the thalweg (Table S7) suggesting faster carbon turnover times, and a larger role in terrestrial carbon processing than would be expected purely based on aerial extent of these nearshore riverbank waters.

Excluding these hot spots of terrestrial carbon inflow and processing from estimates of carbon transport and transfer within river systems adds to existing uncertainties and sampling biases (Drake et al. 2018). Additionally, these are likely hot spots for sedimentation and burial of terrestrial carbon, processes that remain poorly constrained in inland waters (Drake et al. 2018). Future research should pay more attention to these environments, especially in the Arctic where riverbank erosion and in general, terrestrial‐aquatic connectivity is increasing due to permafrost thaw (e.g., Kokelj et al. 2021; Beel et al. 2021). These macro environments may play a disproportionately large role in terrestrial carbon processing along the land‐ocean continuum.

### 4.5 Climate Impact of Abrupt Permafrost Thaw

Our incubations suggest that Duvanny Yar thaw streams emit large amounts of CO_2_ and that these emissions continue after the thaw streams enter the Kolyma River. At the same time, it is likely that large part of permafrost‐derived POC released to the Kolyma River from Duvanny Yar either sinks to the riverbed or dilutes with the Kolyma waters as POC concentration were an order of magnitude lower in outflow waters than in the thaw streams. Measured old ages of riverbed sediments in the Kolyma River (5850–7020 uncalibrated radiocarbon years.; Jong et al. 2023) support deposition to riverbed sediments, however, part of the POC is transported downstream in the river (Keskitalo et al. 2022; Wild et al. 2019). Near‐riverbank environments show higher losses in DOC during whole‐water incubations than those for the Kolyma River thalweg, suggesting that these environments that receive high amounts of sediments via thaw streams emit more GHG than waters in the thalweg of the river. While these macro environments near riverbanks might not cover a large proportion of the river, they increase the overall river GHG emissions. Especially in smaller rivers, their influence might be greater due to their larger proportion of the total river area than in larger rivers, thus their influence should be considered in overall river emission budgets.

While Duvanny Yar may only be one example of a point source of GHG emissions, climate warming is expected to increase the occurrence and expansion of abrupt thaw features (Jorgenson et al. 2006; Kokelj et al. 2021; Schuur et al. 2015). Understanding these features is particularly important as permafrost areas rich in ground ice (like Duvanny Yar) cover only 20% of the landscape, yet they contain ~50% of the total ground OC (Olefeldt et al. 2016; Turetsky et al. 2020). Given the vulnerability and high OC storage of these landscapes, they may become a significant GHG source in the future when contributions of each site are considered across the Arctic (see e.g., Beer et al. 2023 for an estimate of CO_2_ emissions from retrogressive thaw slumps across Siberia).

## 5 Conclusions and Implications

Our data show that thaw streams draining abruptly thawing permafrost at Duvanny Yar have extremely high concentrations of POC, DOC, and DIC compared to the outflow waters in the Kolyma River or the Kolyma River thalweg. Waters in the Kolyma near riverbank environment consists of particles that resemble thaw streams (Pleistocene aged permafrost OC), while the composition of the water itself and the dissolved fractions are more alike to the Kolyma waters (δ^18^O, δ^13^C‐DOC), thus forming macro environments where OC degradation differs from thaw streams and Kolyma River thalweg. These waters should be considered in river basin‐wide studies for boundless estimates of carbon dynamics (Battin et al. 2009). Our whole‐water incubations (including both particles and dissolved fractions) suggest that thaw streams are a source of GHG. Absolute emissions of CO_2_ from thaw streams are orders of magnitude higher than in the outflow waters, however, lower once normalized to gdw of suspended sediment. These CO_2_ emissions are likely driven by microbial degradation of DOC combined with other processes such as mineral weathering and/or chemoautotrophy. Contribution of geogenic processes combined with mineral‐organic matter interactions during whole‐water laboratory incubations complicates the interpretation of carbon dynamics and deciphering sources of GHG emissions from whole‐water incubations. We recommend development of incubation methods that can target the complexity of these different processes during whole‐water incubations that best reflect in situ environment.

## Author Contributions

**Kirsi H. Keskitalo:** conceptualization, data curation, formal analysis, funding acquisition, investigation, methodology, validation, visualization, writing – original draft. **Lisa Bröder:** formal analysis, investigation, methodology, writing – review and editing. **Dirk J. Jong:** formal analysis, investigation, writing – review and editing. **Tommaso Tesi:** formal analysis, writing – review and editing. **Paul J. Mann:** formal analysis, writing – review and editing. **Anna Davydova:** investigation, resources, writing – review and editing. **Nikita Zimov:** investigation, resources. **Negar Haghipour:** formal analysis. **Timothy I. Eglinton:** formal analysis, resources. **Jorien E. Vonk:** conceptualization, formal analysis, funding acquisition, investigation, methodology, project administration, resources, supervision, writing – review and editing.

## Conflicts of Interest

The authors declare no conflicts of interest.

## Supporting information


Data S1.

## Data Availability

The data that support the findings of this study are openly available in the article Supporting Information and in Zenodo at http://doi.org/10.5281/zenodo.14568729.
