# Supplementary material for: Greenhouse Gas Emissions and Lateral Carbon Dynamics at an Eroding Yedoma Permafrost Site in Siberia (Duvanny Yar)
Source: Glob Chang Biol. 2025 Feb 14;31(2):e70071. doi: 10.1111/gcb.70071 (PMC11826377; doi:10.1111/gcb.70071)
Supplement: Supplementary file 1 — Data S1. [file GCB-31-e70071-s001.pdf]

Supporting information for 'Greenhouse gas emissions and lateral carbon dynamics at an eroding Yedoma permafrost site in Siberia (Duvanny Yar)'

Kirsi H. Keskitalo[1,2], Lisa Bröder[2,3], Dirk J. Jong[2], Paul J. Mann[1], Tommaso Tesi[4], Anna Davydova[5], Nikita Zimov[5], Negar Haghipour[3,6], Timothy I. Eglinton[3] and Jorien E. Vonk[2]

[1]Department of Geography and Environmental Sciences, Northumbria University, Newcastle Upon Tyne, UK
[2]Department of Earth Sciences, Vrije Universiteit Amsterdam, Amsterdam, The Netherlands
[3]Department of Earth Sciences, Swiss Federal Institute of Technology, Zürich, Switzerland
[4]National Research Council, Institute of Polar Sciences in Bologna, Italy
[5]Pacific Institute for Geography, Far East Branch, Russian Academy of Sciences, Northeast Science Station, Cherskiy, Republic of Sakha, Yakutia, Russia
[6]Laboratory of Ion Beam Physics, Swiss Federal Institute of Technology, Zürich, Switzerland

Contents:

Supplementary text

Text S1. N_2O during incubations

Text S2. Challenges of filtering high sediment load thaw stream waters

Supplementary figures

Figure S1. Changes in carbon concentrations during whole-water incubations

Figure S2. Flocculation of dissolved organic carbon during thaw stream incubations

Figure S3. Dissolved O_2 saturation percent during incubations of thaw stream and outflow waters

Figure S4. Linear regression on losses of dissolved organic carbon and gains in dissolved inorganic carbon during whole-water incubations

Supplementary tables

Table S1. Welch's t-test results comparing initial conditions in thaw streams and outflow waters

Table S2. Concentrations of dissolved gases in thaw streams

Table S3. Analysis of variance on different parameters during whole-water incubations of thaw stream waters

Table S4. Analysis of variance on different parameters during whole-water incubations of outflow waters

Table S5. The $\Delta^{14}C$ of particulate organic carbon during whole-water incubation of the site OF1HS (outflow waters)

Table S6. Losses/gains of dissolved organic carbon (DOC) and changes in $\delta^{13}C$-DOC in the whole-water (including dissolved and particle fractions) and filtered (only dissolved fraction) incubations of thaw stream waters with and without flocculates

Table S7. Degradation constants for dissolved organic carbon in the whole-water and filtered incubations

Table S8. Analysis of variance test for the separate dissolved organic carbon (DOC only) incubations

Table S9. Welch t-test results on CO_2, CH_4, and N_2O between the initial concentrations and at the end of the incubation concentrations

Table S10. Increase in CO_2 and CH_4 in headspace as CO_2-C and CO_2 and CH_4 per gram dry weight (gdw) of sediment during thaw stream (DY1–DY3) and outflow (OF1HS) incubations

Table S11. Changes in pH during whole-water incubations of thaw stream waters and outflow waters

Table S12. Comparison of CO_2 production per gram dry weight (gdw) of sediment/soil during aerobic incubations between this study and previous studies

Table S13. Welch's t-test results between initial conditions in outflow waters and the Kolyma River thalweg.

Supplementary text:

Text S1. N_2O during incubations:

The headspace N_2O showed increases and decreases during the incubations (Table 3). A previous study by Marushchak et al. (2021) has shown similar results with both increases and decreases in headspace N_2O during incubations of Yedoma soil samples. They suggest that the relatively low emissions might be due to limitation in ammonia (common for high latitude soils), low abundance of ammonia oxidizing bacteria combined with wet (vs dried soils), and aerobic conditions (Marushchak et al., 2021).

Text S2. Challenges of filtering high sediment load thaw stream waters:

While the gains in POC are apparent, the high standard deviations are caused by challenges in filtering these sediment-saturated waters. We were not able to filter the entire incubation samples (only ~1 ml can be filtered at the time, while the samples consisted of 80 ml of particle-rich waters), so we relied on subsamples at each time point. To ensure representative subsampling we shook the samples thoroughly before filtering and used replicates ($n = 4$) per bottle. Thus, for each time point (T_1–T_3) we had two to six replicate bottles (depending how quickly O_2 was utilized) and for each bottle we filtered four replicate subsamples for TSS and POC measurements (Keskitalo et al., 2025). While diluting the samples for the incubations could have been a solution (see e.g., Shakil et al. 2021), these samples would no longer represent the actual thaw stream conditions characterized by an extremely high sediment load.

Figures:

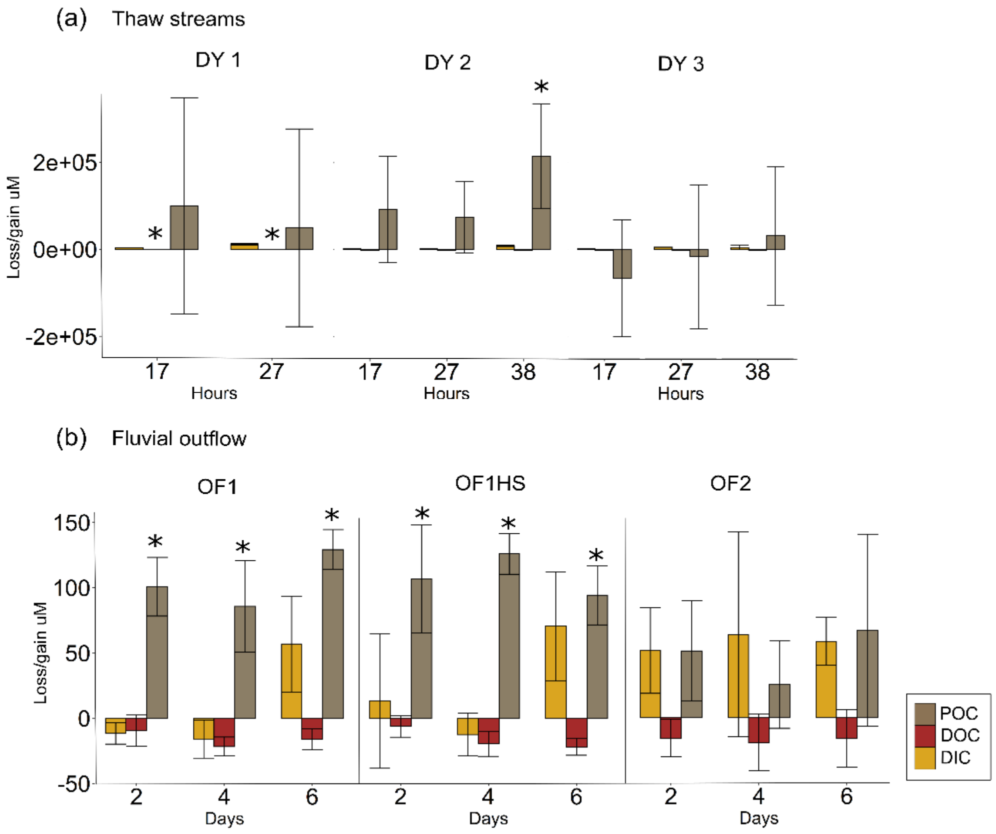

Figure S1. Changes in particulate and dissolved organic carbon (POC and DOC, respectively; mean ± se) and dissolved inorganic carbon (DIC) during whole-water incubations shown as changes from T_0 (non-incubated waters) in (a) thaw streams and (b) fluvial outflow (OF) sites, note that sites OF1 and OF2 are without headspace and OF1HS is with headspace. Thaw stream incubations were all with headspace. The asterisks above the bars indicate significant difference from the initial (T_0) conditions (see Table S7 and S8 for details).

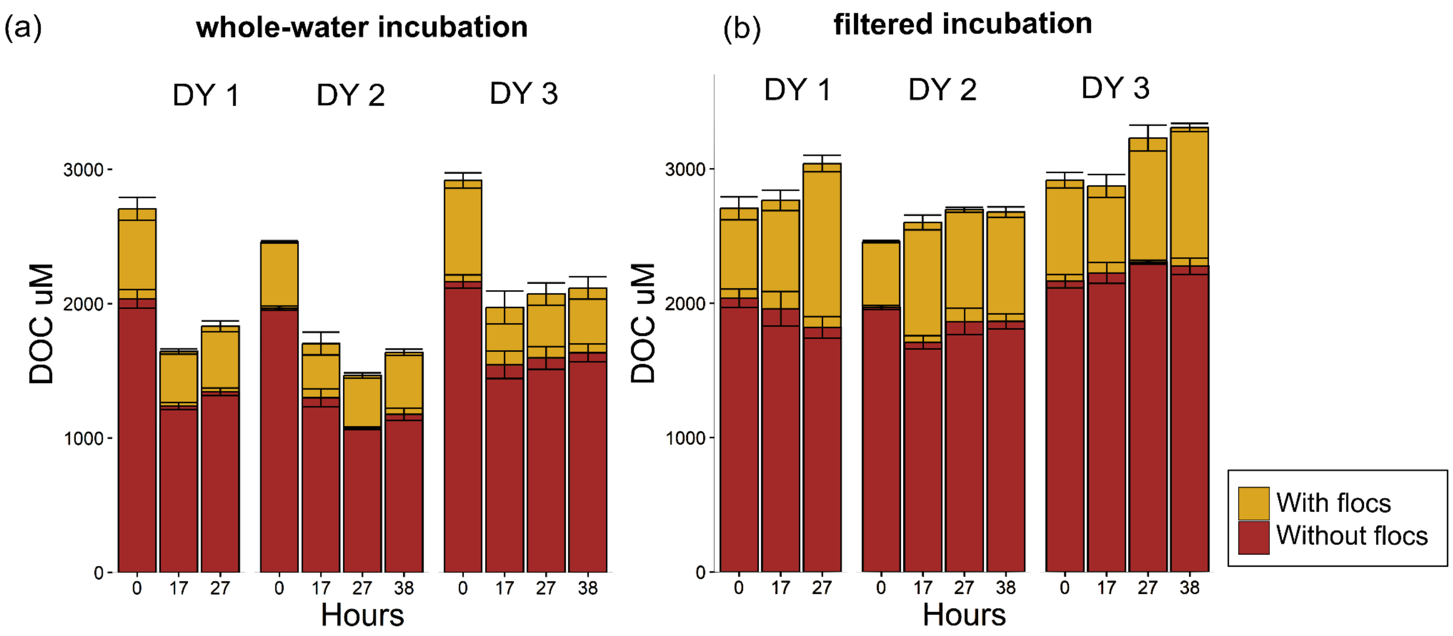

Figure S2. Flocculation of dissolved organic carbon (DOC; mean ± se) in thaw stream incubations. (a) Whole-water incubations with (DOC + flocs) and DOC without flocs. (b) Filtered incubations with (DOC + flocs) and DOC without flocs.

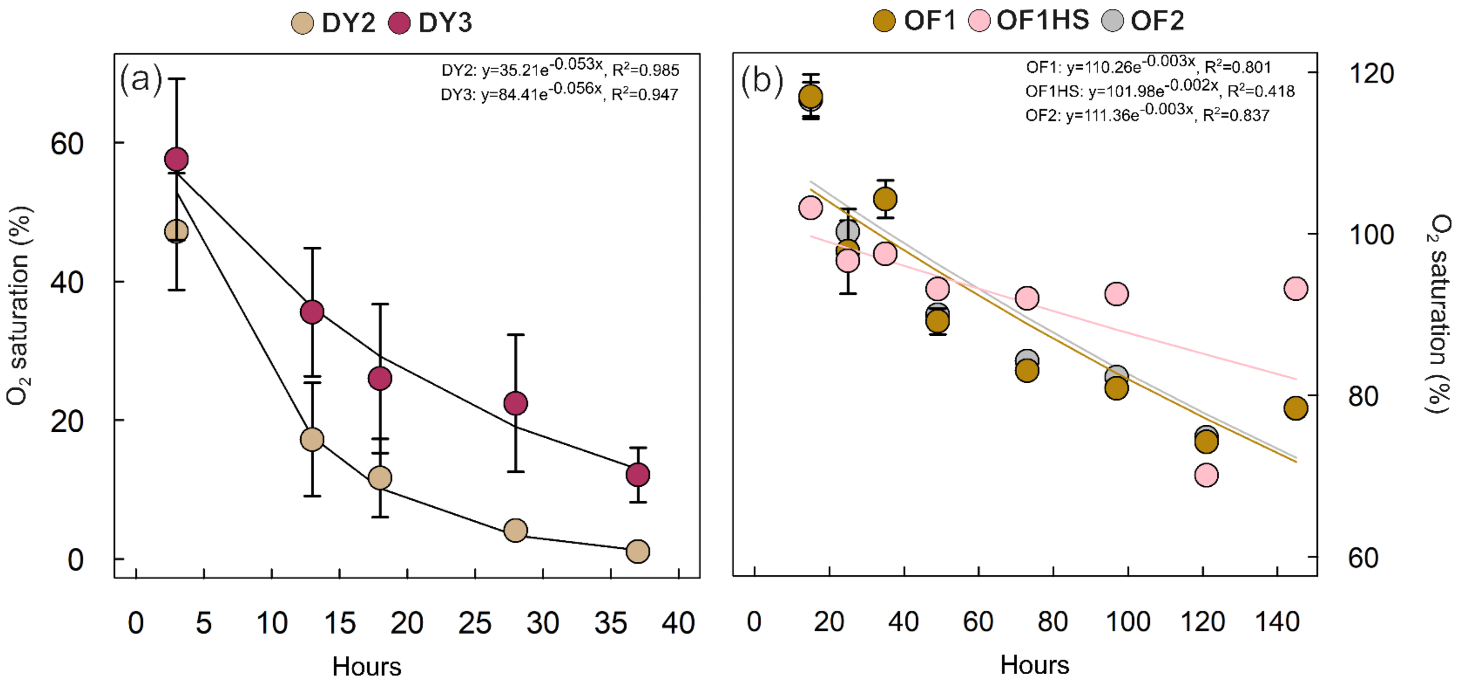

Figure S3. Dissolved O_2 saturation-% (mean ± standard deviation) during incubations of (a) thaw streams and (b) outflow waters. Note that the y-axes are on a different scale. Standard deviations smaller than the symbol are not shown.

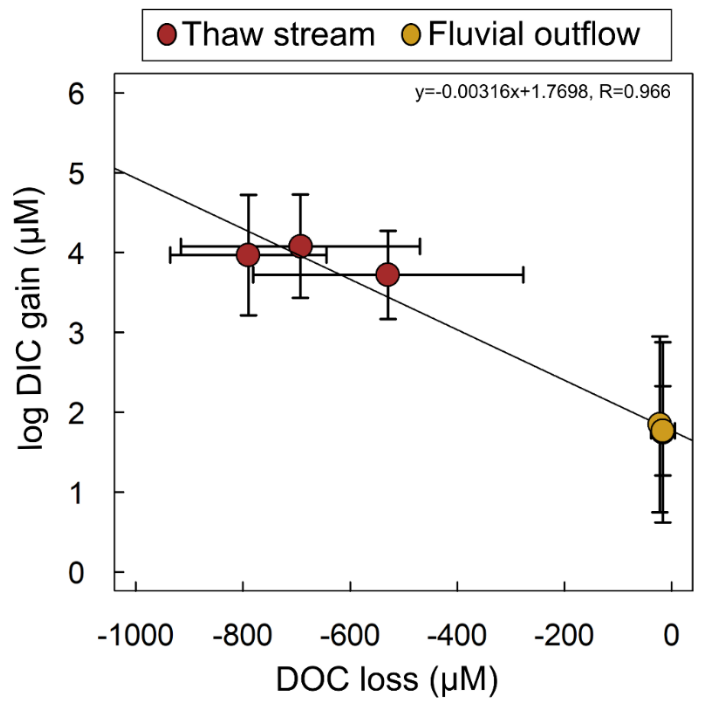

Figure S4. Loss of dissolved organic carbon (DOC) and gain in dissolved inorganic carbon (DIC, log) at the end of the whole-water incubations of thaw streams and outflow waters. The whiskers show standard deviations between replicate samples (n=2–6). The linear regression was statistically significant (R^2=0.966, $F(1,4)$=143.1, $p<0.001$).

Tables:

Table S1. Welch t-test (or Mann Whitney U test when t-test assumptions were not met) results for comparisons of particulate and dissolved organic carbon (POC and DOC, respectively), ratio between POC and total particulate nitrogen (TPN), $\delta^{13}C$ of POC and DOC, $\Delta^{14}C$-POC, $\delta^{18}O$ and dissolved O_2 between thaw streams and outflow waters. We did not have enough DIC or $\delta^{13}C$-DIC data to test their statistical difference between thaw streams and outflow sites. Statistically significant results are highlighted in bold.

	**POC**	**POC-%**	**$\delta^{13}C$-POC**	**$\Delta^{14}C$-POC**	**POC/TPN**	**TSS**	**DOC**	**$\delta^{13}C$-DOC**	**$\delta^{18}O$**	**O_2**
t or Z-statistic	**t(19)=17.092**	**Z=8**	t(19)=1.4499	t(2.92)=0.6654	Z=42	**Z=152**	**Z=54**	**t(10.478)=16.211**	**t(2.1263)=-39.179**	**t(21.291)=-13.993**
p-value	**<0.001**	**<0.001**	0.1627	0.5545	>0.05	**<0.001**	**<0.001**	**<0.001**	**<0.001**	**<0.001**

Table S2. Dissolved gases (CO_2, CH_2, and N_2O) in thaw stream sites DY1 (n=1), DY2 (n=2), DY3 (n=2). Also shown dissolved gases measured in the Kolyma River thalweg (n=6) for comparison and atmosphere (n=9) measured at Duvanny Yar. These data are not available for the outflow waters.

**Site**	**CO_2 (ppm)**	**CH_4 (ppm)**	**N_2O (ppm)**
DY1	2370	2.6	0.8
DY2	2350±250	2.07±0.2	0.8±0.1
DY3	2370±301	3.2±0.3	0.3±0.02
Kolyma*	568±52	4.12±0.3	0.23±0.02
Atmosphere	413±6.4	1.6±0.2	0.24±0.02

*Unpublished data shown here for comparison.

Table S3. Analysis of variance (ANOVA) or Kruskal-Wallis test results of the changes in particulate and dissolved organic carbon (POC and DOC, respectively), POC-% (i.e., amount of OC from total suspended solids, TSS), total particulate nitrogen (TPN), TSS and δ^{13}C-OC during thaw stream incubations at sites DY1, DY2 and DY3. Also shown are results for DOC and δ^{13}C-DOC of the flocculates. All significant results are shown in bold. The abbreviation n/a refers to 'not applicable'. For the ANOVA results of the outflow sites, see Table S4.

Site	TSS	POC	POC-%	δ^{13}C-POC	TPN	DOC	δ^{13}C-DOC	DOC floc	δ^{13}C-DOC floc
**DY1**	H(2)= 3.1176 p>0.05	F(2,13)= 0.766 p=0.485	H(2)= 2.0129 p=0.366	H(2)= 0.763 p=0.683	F(2,13)= 0.818 p=0.463	**F(2,8)= 38.85 p<0.001**	F(2,9)= 0.391 p>0.05	**H(2)= 8.0485 p<0.05**	**H(2)= 6.0641 p<0.05**
T0–T1	n/a	n/a	n/a	n/a	n/a	**p<0.001**	n/a	**p=0.014**	p=0.92
T0–T2	n/a	n/a	n/a	n/a	n/a	**p<0.001**	n/a	p=0.249	**p=0.047**
T0–T3	n/a	n/a	n/a	n/a	n/a	n/a	n/a	p=0457	p=0.64
**DY2**	**F(3,11)= 3.845 p=0.042**	**F(3,11)= 14.74 p<0.001**	**H(3)= 9.5833 p=0.022**	**F(3,11)= 6.073 p=0.01**	**F(3,11)= 9.979 p<0.01**	H(3)= 7.641 p>0.05	**F(3,8)= 6.311 p<0.05**	H(3)= 7.5 p>0.05	**F(3,8)= 6.311 p<0.05**
T0–T1	n/a	**p=0.025**	**p=0.040**	**p=0.042**	p=0.239	n/a	p=0.09	n/a	p=0.09
T0–T2	n/a	p=0.260	p=0.268	**p=0.026**	p=0.634	n/a	p=0.09	n/a	p=0.09
T0–T3	n/a	**P<0.001**	p=0.271	p=0.059	**p=0.001**	n/a	**p=0.012**	n/a	**p=0.012**
**DY3**	**H(3)= 11.438 p<0.01**	F(3,11)= 0.861 P>0.05	**F(3,11)= 15.62 p<0.001**	F(3,11)= 0.477 p=0.705	F(3,11)= 1.835 p=0.199	**F(3,8)= 4.705 p=0.035**	F(3,8)= 3.7 p>0.05	**F(3,8)= 7.816 p<0.01**	F(3,8)= 3.271 p>0.05
T0–T1	**p<0.01**	n/a	**p<0.001**	n/a	n/a	**p=0.043**	n/a	**p=0.01**	n/a
T0–T2	p=0.240	n/a	**p=0.035**	n/a	n/a	p=0.099	n/a	**p=0.04**	n/a
T0–T3	p=1.000	n/a	**p=0.020**	n/a	n/a	p=0.064	n/a	**p=0.02**	n/a

Table S4. Analysis of variance (ANOVA) or Kruskal-Wallis test (used when data did not fulfil assumptions of ANOVA) results of the changes in particulate and dissolved organic carbon (POC and DOC, respectively), POC-% (i.e., amount of OC from total suspended solids, TSS), total particulate nitrogen (TPN), TSS, δ^{13}C-OC and Δ^{14}C-POC during incubations of outflow waters at sites OF1, OF1HS (with headspace) and OF2. All significant results are shown in bold. The abbreviation n/a refers to 'not applicable'. For the ANOVA results of the thaw streams, see Table S3.

Site	TSS	POC	POC-%	δ^{13}C-POC	Δ^{14}C-POC	TPN	DOC	δ^{13}C-DOC
**OF1**	H(3)= 8.1099 p=0.043	**F(3,9)= 21.98 p<0.001**	F(3,9)= 2.421 p>0.05	F(3,9)= 2.538 p=0.122	n/a	H(3)= 8.2857 p=0.040	**F(3,8)= 5.156 p=0.028**	F(3,8)= 3.203 p=0.084
T0–T1	n/a	**p<0.001**	n/a	n/a	n/a	n/a	p=0.414	n/a
T0–T2	n/a	**p<0.001**	n/a	n/a	n/a	n/a	**p=0.024**	n/a
T0–T3	n/a	**p<0.01**	n/a	n/a	n/a	n/a	p=0.095	n/a
**OF1HS**	**F(3,9)= 7.887 p<0.01**	**F(3,9)= 27.6 p<0.001**	H(3)= 4.2582 p>0.05	H(3)= 1.7967 p=0.616	n/a	**F(3,9)= 22.29 p=0.001**	**H(3)= 7.923 p=0.048**	F(3,8)= 1.197 p=0.371
T0–T1	**p=0.012**	**p<0.001**	n/a	n/a	n/a	**p<0.001**	n/a	n/a
T0–T2	**p=0.021**	**p<0.01**	n/a	n/a	n/a	**p<0.01**	n/a	n/a
T0–T3	**p=0.023**	**p<0.001**	n/a	n/a	n/a	**p<0.001**	n/a	n/a
**OF2**	**F(3,9)= 4.233 p=0.04**	H(3)= 5.006 P>0.05	H(3)= 2.5879 p>0.05	H(3)= 4.302 p=0.231	H(3)= 9.011 p=0.029	F(3,11)= 1.293 p=0.335	F(3,9)= 2.058 p=0.176	**F(3,9)= 6.864 p=0.011**
T0–T1	p=0.209	n/a	n/a	n/a	n/a	n/a	n/a	**p=0.007**
T0–T2	p=0.262	n/a	n/a	n/a	n/a	n/a	n/a	p=0.245
T0–T3	**p=0.030**	n/a	n/a	n/a	n/a	n/a	n/a	p=0.082

Table S5. The $\Delta^{14}C$ and uncalibrated ages of particulate organic carbon (POC) during a six-day whole-water incubation at the site OF2. For the timepoint zero (zero days) n=4 and for all the other timepoints (days 2–6) n=3.

Time (days)	$\Delta^{14}C$ (‰)	Age (uncalibrated yrs)	ETH ID
0	-853±84	15 370±723	101956.1.1–101959.1.1.
2	-842±90	14 740±665	101947.1.1–101949.1.1.
4	-843±85	14 830±637	101950.1.1–101952.1.1.
6	-854±90	15 380±650	101953.1.1–101955.1.1.

Table S6. Losses/gains of dissolved organic carbon (DOC in µM and %) and changes in $\delta^{13}C$-DOC in the whole-water (including dissolved and particle fractions) and filtered (only dissolved fraction) incubations at thaw streams DY1, DY2 and DY3. Losses/gains are shown with and without the flocculates/flocs.

		Whole-water incubation no flocs			Whole-water incubation with flocs		Filtered incubation no flocs			Filtered incubation with flocs	
Site	Hours (days)	µM	%	$\delta^{13}C$ (‰)	µM	%	µM	%	$\delta^{13}C$ (‰)	µM	%
DY1	27 (~1,1)	-692	-34	+0.36	-874±281	-32	-217±315	-11	+1.15	+380±313	+40
DY2	38 (~1.6)	-790	-40	-0.05	-821±73	-33	-102±175	-5	+0.85	+220±116	+9
DY3	38 (~1.6)	-530	-24	+0.74	-800±302	-27	+111±235	+5	+0.78	+390±196	+13

Table S7. Degradation constants (*k*, day^{-1}) for dissolved organic carbon (DOC) in the whole-water and filtered incubations. For the thaw streams whole-water incubations n=3 and for filtered incubations n=2. For incubations of outflow waters, n=3 in whole-water incubations and n=1 in filtered incubations and for the Kolyma waters (sampled in the thalweg) n=3 for whole-water incubations and n=2 for filtered incubations.

Site	Whole-water incubations	Filtered incubations
Thaw streams	-0.29 ± 0.10	-0.067 ± 0.05
Outflow	-0.01 ± 0.002	-0.03
Kolyma*	-0.006 ± 0.002	-0.012 ± 0.002

*The Kolyma data are from Keskitalo et al. (2022).

Table S8. Analysis of variance (ANOVA) or Kruskal-Wallis test of the separate dissolved organic carbon (DOC only) incubations. Separate testing was performed for the DOC in the flocculated material in the thaw streams (no flocculation occurred in the outflow waters). The abbreviation n/a refers to 'not applicable'.

	**DOC**	**δ^{13}C-DOC**	**DOC floc**	**δ^{13}C-DOC floc**
**DY1**	F(2,9)=0.699 p>0.05	**H(2)=6.5759 p<0.05**	F(2,9)=3.158 p>0.05	F(2,9)=1.759 p>0.05
T0–T1	n/a	p=0.639	n/a	n/a
T0–T2	n/a	**p=0.032**	n/a	n/a
T0–T3	n/a	p=0.799	n/a	n/a
**DY2**	F(3,8)=3.127 p>0.05	H(3)=3.7179 p>0.05	F(3,8)=2.85 p>0.05	F(3,8)=1.427 p>0.05
T0–T1	n/a	n/a	n/a	n/a
T0–T2	n/a	n/a	n/a	n/a
T0–T3	n/a	n/a	n/a	n/a
**DY3**	F(3,8)=0.757 p>0.05	F(3,8)=1.632 P>0.05	F(3,8)=4.064 p>0.05	H(3)=4.6987 p>0.05
T0–T1	n/a	n/a	n/a	n/a
T0–T2	n/a	n/a	n/a	n/a
T0–T3	n/a	n/a	n/a	n/a
**OF1**	**F(3,8)=5.252 p<0.05**	F(3,8)=0.415 p>0.05	n/a	n/a
T0–T1	p=0.080	n/a	n/a	n/a
T0–T2	p=0.084	n/a	n/a	n/a
T0–T3	**p=0.024**	n/a	n/a	n/a
**OF2**	F(3,9)=1.125 p>0.05	F(3,9)=0.566 p>0.05	n/a	n/a
T0–T1	n/a	n/a	n/a	n/a
T0–T2	n/a	n/a	n/a	n/a
T0–T3	n/a	n/a	n/a	n/a

Table S9. Welch t-test or Mann-Whiteny U test results on CO_2, CH_4, and N_2O gases between the initial (T_0) concentrations and at the end of the incubation (T_3). For the site DY2, time points T_2 and T_3 were combined as for T_3 the number of samples was low (n = 2). For all sites T_0 n = 3, for thaw streams T_3 n = 4. For outflow (OF1HS) T_3 n = 3.

Site	CO_2	CH_4	N_2O
DY1	**t(3)=-42.009, p <0.001**	**t(3.3)=-17.735, p <0.001**	**t(3.9)=10.243, p <0.001**
DY2	**t(3)=-8.0602, p <0.01**	t(3)=-2.3316, p >0.05	**t(3.6)=11.735, p <0.001**
DY3	**t(3)=-3.6538, p <0.05**	t(3.2)=-1.7462, p >0.05	z=3, p>0.05
OF1HS	**t(2.42)=-21.849, p <0.001**	**t(3.3)=-17.741, p <0.001**	z=4, p>0.05

Table S10. Increase in CO_2 and CH_4 in headspace as CO_2-C and CO_2 and CH_4 per gram dry weight (gdw) day^{-1} of sediment during thaw stream (DY1–DY3) and outflow (OF1HS) incubations.

Site	Time (hours)	CO_2 (mg CO_2-C g sed)	CO_2 (ug CO_2 gdw^{-1} day^{-1})	CH_4 (µg CO_2-C g sed)	CH_4 (ug CH_4 gdw^{-1} day^{-1})
DY1	27	+0.45 ± 0.02	+33.66 ± 1.6	+0.04 ± 0.004	+0.003 ± 0.0002
DY2	36	+0.27 ± 0.10	+13.96 ± 5.1	+0.02 ± 0.02	+0.001 ± 0.0001
DY3	36	+0.22 ± 0.12	+11.65 ± 6.2	+0.01 ± 0.004	+0.001 ± 0.0005
OF1HS	144	+38.4 ± 1.3	+533 ± 18	+111 ± 7	+8.216 ± 0.521

Table S11. Changes in pH during whole-water incubations of thaw stream waters (DY1–DY3) and in outflow waters (OF1, OF1HS, OF2). In brackets is shown the number of replicates (n). The abbreviation n/a refers to ‘not applicable’.

	DY1	DY2	DY3	OF1	OF1HS	OF2
T0	8.16 (1)	8.03 (1)	8.08 (1)	7.73 (1)	7.73 (1)	7.75 (1)
T1	8.02 ± 0.09 (2)	8.14 ± 0.11 (4)	8.19 ± 0.06 (3)	7.59 ± 0.08 (3)	7.72 ± 0.07 (3)	7.61 ± 0 (3)
T2	8.05 ± 0.09 (6)	7.99 ± 0.04 (2)	8.01 ± 0.01 (2)	7.49 ± 0.05 (3)	7.55 ± 0.12 (3)	7.41 ± 0.12 (3)
T3	n/a	8.05 ± 0.05 (3)	8.17 ± 0.12 (4)	7.49 ± 0.09 (3)	7.62 ± 0.04 (3)	7.46 ± 0.10 (3)

Table S12. Production of CO_2 per gram dry weight (gdw) of sediment/soil during aerobic incubations ± standard deviation (std, given when available). The abbreviation PF=permafrost, AL=active layer, sed= sediment, SW=seawater. In Lee et al. (2012) ToolikKarst refers to thermokarst gully near Toolik Lake in Alaska, EML refers to Eight Mile Lake (Alaska) and Min/Mod/Ext to minimal/modest/extensive degree of thaw. Their Itkillik site 1 is at acidic tundra while Itkillik 2 is at non-acidic tundra. The samples in Lee et al. (2012) were collected within one meter depth except for Fox (Alaska) and Zelenyi Mys (Siberia) that were collected in 10 m depth. In Knoblauch et al. (2021), WI=Weichselian interstadial (sampled at depths of 3.3–11.3 m) and WS=Weichselian stadial (sampled at depths of 16–22.4 m). In the same study, the Holocene samples were collected in depths between 0.7 and 4.3 m (Kurungnakh/Samoylov). In Faucherre et al. (2021), ALns= active layer near-surface (sampled at depth 3.1 ± 2.2 cm), ALss= active layer subsurface (sampled at depth 22.1 ± 11.6 cm) and Alp = alpine.

Study	Time (days)	temp (°C)	µg CO_2 gdw day^{-1}	Type	Sample amount (g)	Material	Incubation conditions	Location
Tanski et al 2021	60	4	1.94±0.14	mud	20 g	sed	ambient	Herschel/Qikiqtaruk
Tanski et al 2021	60	4	1.94±0.04	mud+SW	20 g	sed	seawater	Herschel/Qikiqtaruk
Tanski et al 2021	60	4	5.55±1.25	Cliff	20 g	sed	ambient	Herschel/Qikiqtaruk
Tanski et al 2021	60	4	7.91±0.56	Cliff + SW	20 g	sed	seawater	Herschel/Qikiqtaruk
Tanski et al 2019	120	4	2.98±0.69	PF org +SW	20 g	sed	seawater	Herschel/Qikiqtaruk
Tanski et al 2019	120	4	2.50±0.42	PF min + SW	20 g	sed	seawater	Herschel/Qikiqtaruk
Tanski et al 2019	120	16	3.82±0.49	PF org +SW	20 g	sed	seawater	Herschel/Qikiqtaruk
Tanski et al 2019	120	16	5.13±0.76	PF min + SW	20 g	sed	seawater	Herschel/Qikiqtaruk
this study	6	15	533±18	outflow	<1 g	sed	fresh water	Duvanny Yar
this study	1.1	15	33.66±1.6	thaw stream	60 g	sed	fresh water	Duvanny Yar
this study	1.6	15	13.96±5.1	thaw stream	60 g	sed	fresh water	Duvanny Yar
this study	1.6	15	11.65±6.2	thaw stream	60 g	sed	fresh water	Duvanny Yar
Knoblauch et al 2021	18	4	12.23	slump floor	20 g	soil	soil moisture	Kurungnakh
Knoblauch et al 2021	18	4	44.35	slump floor	20 g	soil	soil moisture	Kurungnakh
Knoblauch et al 2021	18	4	35.51	slump floor	20 g	soil	soil moisture	Kurungnakh
Knoblauch et al 2021	18	4	34.89	slump floor	20 g	soil	soil moisture	Kurungnakh
Knoblauch et al 2021	18	4	29.04	thermokarst mound	20 g	soil	soil moisture	Kurungnakh
Knoblauch et al 2021	18	4	15.22	slump floor	20 g	soil	soil moisture	Kurungnakh
Knoblauch et al 2021	18	4	33.92	slump floor	20 g	soil	soil moisture	Kurungnakh
Knoblauch et al 2021	18	4	36.12	slump floor	20 g	soil	soil moisture	Kurungnakh
Knoblauch et al 2021	18	4	19.71	thermokarst mound	20 g	soil	soil moisture	Kurungnakh
Lee et al 2012	500	15	0.44	glacial drift	50-100 g	soil	soil moisture	Itkillik 1
Lee et al 2012	500	15	0.57±0.01	glacial drift	50-100 g	soil	soil moisture	Itkillik 2
Lee et al 2012	500	15	0.30±0.05	glacial drift	50-100 g	soil	soil moisture	Sagavanirkotk
Lee et al 2012	500	15	0.48±0.11	mineral	50-100 g	soil	soil moisture	EMLMin
Lee et al 2012	500	15	0.24±0.07	mineral	50-100 g	soil	soil moisture	EMLMod
Lee et al 2012	500	15	0.48±0.12	mineral	50-100 g	soil	soil moisture	EMLExt
Lee et al 2012	500	15	0.22±0.14	glacial drift	50-100 g	soil	soil moisture	ToolikKarst
Lee et al 2012	500	15	0.18±0.03	Yedoma	50-100 g	soil	soil moisture	Fox
Lee et al 2012	500	15	0.28±0.05	Yedoma	50-100 g	soil	soil moisture	Zelenyi Mys
Lee et al 2012	500	15	6.59±1.58	organic	50-100 g	soil	soil moisture	EMLMin
Lee et al 2012	500	15	7.49±2.84	organic	50-100 g	soil	soil moisture	EMLMod
Lee et al 2012	500	15	11.97±1.49	organic	50-100 g	soil	soil moisture	EMLExt
Knoblauch et al 2012	1200	4	2.27±0.68	Holocene PF	20 g	soil	soil moisture	Kurungnakh/ Samoylov
Knoblauch et al 2012	1200	4	2.14±0.83	Pleistocene PF (WS)	20 g	soil	soil moisture	Kurungnakh
Knoblauch et al 2012	1200	4	1.98±0.96	Pleistocene PF (WI)	20 g	soil	soil moisture	Kurungnakh
Faucherre et al 2018	343	5	2.01±1.7	AL ns	3-5 g	soil	soil moisture	Svalbard
Faucherre et al 2018	343	5	0.10±0.15	AL ss	3-5 g	soil	soil moisture	Svalbard
Faucherre et al 2018	343	5	0.10±0.05	PF	3-5 g	soil	soil moisture	Svalbard
Faucherre et al 2018	343	5	2.38±2.21	Holocene AL ns	3-5 g	soil	soil moisture	Lena Delta
Faucherre et al 2018	343	5	0.29±0.34	Holocene AL ss	3-5 g	soil	soil moisture	Lena Delta
Faucherre et al 2018	343	5	0.27±0.22	Holocene PF	3-5 g	soil	soil moisture	Lena Delta
Faucherre et al 2018	343	5	3.35±1.70	Pleistocene AL ns	3-5 g	soil	soil moisture	Lena Delta
Faucherre et al 2018	343	5	0.24±0.24	Pleistocene AL ss	3-5 g	soil	soil moisture	Lena Delta
Faucherre et al 2018	343	5	0.22±0.15	Pleistocene PF	3-5 g	soil	soil moisture	Lena Delta
Faucherre et al 2018	343	5	2.09±2.57	Alp	3-5 g	soil	soil moisture	Abisko (Stordalen)

Table S13. Welch t-test (or Mann Whitney U test when t-test assumptions were not met) results for comparisons of total suspended solids (TSS), particulate and dissolved organic carbon (POC and DOC, respectively), ratio between POC and total particulate nitrogen (TPN), δ^{13}C-OC, Δ^{14}C-POC and δ^{18}O between outflow waters and the Kolyma thalweg (Kolyma thalweg data from Keskitalo et al., 2022). We did not have enough DIC or δ^{13}C-DIC data to test their statistical difference between thaw streams and outflow sites. Statistically significant results are highlighted in bold.

	**POC**	**POC-%**	**δ^{13}C-POC**	**Δ^{14}C-POC**	**POC/TPN**	**TSS**	**DOC**	**δ ^{13}C-DOC**	**δ^{18}O**
t or Z-statistic	**t(7.2523)=16.508**	**t(7.2821)=-4.9795**	**Z=80**	**t(4.4466)=-34.702**	**t(10.474)=-5.0231**	**t(7.9152)=22.727**	**t(11.993)=-7.8819**	t(7.8353)=0.64236	Z=12
p-value	**<0.001**	**<0.001**	**<0.001**	**<0.001**	**<0.001**	**<0.001**	**<0.001**	>0.05	>0.05

References:

Keskitalo, K. H., Bröder, L., Jong, D., Zimov, N., Davydova, A., Davydov, S., Tesi, T., Mann, P. J., Haghipour, N., Eglinton, T. I. and Vonk, J. E. (2025) Biogeochemistry and greenhouse gases at an eroding Yedoma permafrost site in Siberia (Duvanny Yar). Zenodo. https://doi.org/10.5281/zenodo.14568729

Marushchak, M. E., Kerttula, J., Diáková, K., Faguet, A., Gil, J., Grosse, G., Knoblauch, C., Lashchinskiy, N., Martikainen, P. J., Morgenstern, A., Nykamb, M., Ronkainen, J. G., Siljanen, H. M. P., van Delden, L., Voigt, C., Zimov, N., Zimov, S. and Biasi, C. (2021) Thawing Yedoma permafrost is a neglected nitrous oxide source. Nat. Commun., 12, https://doi:10.1038/s41467-021-27386-2

Shakil, S., Tank, S., Vonk, J. and Zolkos, S. (2022) Low biodegradability of particulate organic carbon mobilized from thaw slumps on the Peel Plateau, NT, and possible chemosynthesis and sorption effects. Biogeosciences, 19, 1871–1890, https://doi.org/10.5194/bg-19-1871-2022
